# Predicting secondary organic aerosol phase state and viscosity and its effect on multiphase chemistry in a regional-scale air quality model

**DOI:** 10.5194/acp-20-8201-2020

**Published:** 2020-07-16

**Authors:** Ryan Schmedding, Quazi Z. Rasool, Yue Zhang, Havala O. T. Pye, Haofei Zhang, Yuzhi Chen, Jason D. Surratt, Felipe D. Lopez-Hilfiker, Joel A. Thornton, Allen H. Goldstein, William Vizuete

**Affiliations:** 1 Department of Environmental Science and Engineering, The University of North Carolina at Chapel Hill, Chapel Hill, NC 27516, USA; 2 Office of Research and Development, Environmental Protection Agency, Research Triangle Park, Durham, NC 27709, USA; 3 Department of Chemistry, University of California at Riverside, Riverside, CA 92521, USA; 4 Aerodyne Research, Inc., Billerica, MA 01821, USA; 5 Department of Atmospheric Sciences, University of Washington, Seattle, WA 98195, USA; 6 Department of Environmental Science, Policy, and Management, University of California, Berkeley, CA 94720, USA; 7 Department of Civil and Environmental Engineering, University of California, Berkeley, CA 94720, USA

## Abstract

Atmospheric aerosols are a significant public health hazard and have substantial impacts on the climate. Secondary organic aerosols (SOAs) have been shown to phase separate into a highly viscous organic outer layer surrounding an aqueous core. This phase separation can decrease the partitioning of semi-volatile and low-volatile species to the organic phase and alter the extent of acid-catalyzed reactions in the aqueous core. A new algorithm that can determine SOA phase separation based on their glass transition temperature (*T*_g_), oxygen to carbon (O : C) ratio and organic mass to sulfate ratio, and meteorological conditions was implemented into the Community Multiscale Air Quality Modeling (CMAQ) system version 5.2.1 and was used to simulate the conditions in the continental United States for the summer of 2013. SOA formed at the ground/surface level was predicted to be phase separated with core–shell morphology, i.e., aqueous inorganic core surrounded by organic coating 65.4 % of the time during the 2013 Southern Oxidant and Aerosol Study (SOAS) on average in the isoprene-rich southeastern United States. Our estimate is in proximity to the previously reported ~ 70 % in literature. The phase states of organic coatings switched between semi-solid and liquid states, depending on the environmental conditions. The semi-solid shell occurring with lower aerosol liquid water content (western United States and at higher altitudes) has a viscosity that was predicted to be 10^2^–10^12^ Pa s, which resulted in organic mass being decreased due to diffusion limitation. Organic aerosol was primarily liquid where aerosol liquid water was dominant (eastern United States and at the surface), with a viscosity < 10^2^ Pa s. Phase separation while in a liquid phase state, i.e., liquid–liquid phase separation (LLPS), also reduces reactive uptake rates relative to homogeneous internally mixed liquid morphology but was lower than aerosols with a thick viscous organic shell. The sensitivity cases performed with different phase-separation parameterization and dissolution rate of isoprene epoxydiol (IEPOX) into the particle phase in CMAQ can have varying impact on fine particulate matter (PM_2.5_) organic mass, in terms of bias and error compared to field data collected during the 2013 SOAS. This highlights the need to better constrain the parameters that govern phase state and morphology of SOA, as well as expand mechanistic representation of multiphase chemistry for non-IEPOX SOA formation in models aided by novel experimental insights.

## 1 Introduction

Particulate matter (PM) is one of six criteria pollutants regulated by the United States Environmental Protection Agency (EPA)’s National Ambient Air Quality Standards (NAAQS), established by the 1970 Clean Air Act. There are two categories of PM regulated by NAAQS: fine PM (PM_2.5_) with particle diameter less than 2.5 μm and coarse PM (PM_10_) with particle diameter up to 10 μm. PM has adverse effects on the global climate (Carslaw et al., 2013; Grandey et al., 2018; L. A. Lee et al., 2016; Regayre et al., 2015). PM_2.5_ also represents a substantial public health risk due to its association with increased overall mortality, due to cardiorespiratory diseases (Hwang et al., 2017; Jaques and Kim, 2000; Zanobetti and Schwartz, 2009). It has been estimated that 20 %–60 % of PM_2.5_ are comprised of organic aerosols (OAs) (Docherty et al., 2008). These pollutant species are either directly emitted primary organic aerosols (POAs) or secondary organic aerosols (SOAs), which form when volatile organic compounds (VOCs) undergo chemical reactions that reduce their volatility to the point that they partition into the aerosol phase (Zhang et al., 2007) or react heterogeneously with the existing particles (Riva et al., 2019). Studies have found that SOA tends to form the bulk of observed OA around the world (Nozière et al., 2015). The VOCs that form SOA may be either from biogenic or anthropogenic sources and can vary both spatially and temporally to areas as confined as the community level (Yu et al., 2014).

Recent studies have shown that SOA may undergo phase separation under atmospherically relevant conditions resulting in different morphologies. These observations have included a “partially engulfed” organic–inorganic morphology; an emulsified or “island” morphology, where discrete pockets of SOA dot a larger inorganic particle; and a “core–shell” morphology, characterized by an organic-rich outer “shell” and aqueous inorganic “core” (Freedman, 2017; O’Brien et al., 2015; Price et al., 2015; Renbaum-Wolff et al., 2016; Song et al., 2015, 2016; You et al., 2012; Y. Zhang et al., 2015, 2018). Pye et al. (2018) applied the Aerosol Inorganic-Organic Mixtures Functional groups Activity Coefficients (AIOMFAC) model (Zuend et al., 2008) to predict the thermodynamic favorability of phase separation in SOA using a box model and found that aerosols over the southeastern United States may be phase separated as frequently as 70 % of the time. Pye et al. (2017) used the ratio of organic matter to organic carbon (OM : OC) and the ambient relative humidity (RH) to predict phase-separation frequencies. They found that phase separation was common at lower RH in urban areas with low OM : OC, but lower phase-separation frequencies in rural areas were attributed to increasing OM : OC except for late mornings when phase-separation frequency increased due to low RH.

When aerosols form a core–shell morphology, experimentally observed viscosities of the outer organic-rich shell and inner electrolyte-rich core have been shown to differ by up to 3 orders of magnitude, resulting in possible diffusion limitations on reactive uptake (Ullmann et al., 2019). It has also been shown that the viscosity of the organic-rich shell and subsequently diffusivity of gaseous particles through it (*D*_org_) may vary as a function of SOA composition (Grayson et al., 2017). Laboratory experiments have been conducted to measure the viscosity of SOA using poke-flow and bead mobility techniques (Reid et al., 2018; Renbaum-Wolff et al., 2013; Song et al., 2015, 2016). These studies have found that SOAs formed from anthropogenic precursors, such as benzene, toluene and xylene, have similar *D*_org_ values in the realm of 10^−14^–10^−16^m^2^s^−1^ (Grayson et al., 2016; Song et al., 2015, 2016, 2018). Similar studies on biogenic SOA comprised of *α*-pinene oxidation products found that its measured viscosities and calculated diffusion coefficients are lower than those of anthropogenic SOA by as much as 2 orders of magnitude in comparable conditions (Song et al., 2016, 2018; Zhang et al., 2015).

The most abundantly emitted biogenic VOC is isoprene (2-methyl-1,3-butadiene), with average annual global emissions totaling approximately 500–750 TgCyr^−1^ (Guenther et al., 2006; Liao et al., 2015). Isoprene is known to react with hydroxyl (OH) radicals under low-NO*_x_* (= NO + NO_2_) conditions to form isoprene hydroxyhydroperoxides (ISOPOOH) (Jacobs et al., 2014; Krechmer et al., 2015). If the reaction pathway continues with OH, ISOPOOH will react again to form isoprene epoxydiol (IEPOX) (Bates et al., 2014; Paulot et al., 2009; Surratt et al., 2010). It is possible for IEPOX to form products with sufficiently low volatility to form SOA via a reactive uptake onto acidified sulfate seed particles (Bondy et al., 2018; Surratt et al., 2006, 2007, 2010). IEPOX-derived SOA have been observed to account for up to 36 % of biogenic SOA in the southeastern United States during the summer (Budisulistiorini et al., 2016). Given the importance of this pathway, there has been increased focus on the phase state of particles and its impact on reactive uptake (Budisulistiorini et al., 2017).

Prior measurements of isoprene-derived SOA suggested that it would not be viscous enough to exhibit diffusion limitations. There is still much uncertainty with these measurements as those particles are mainly formed through nucleation of semi-volatile species (Song et al., 2015). IEPOX-derived SOA in the southeastern United States is found to exhibit higher volatility than the remaining bulk OA, with saturation vapor pressures for IEPOX-derived SOA being 2 to 8 orders of magnitude larger than the remaining bulk OA. However, IEPOX-derived SOA has a low overall volatility, with evaporation timescales > 100 h under atmospherically relevant conditions (Lopez-Hilfiker et al., 2016). Specifically, acid-driven multiphase chemistry of IEPOX with inorganic sulfate aerosol results in a significant yield of organosulfates that have potentially higher viscosities (Riva et al., 2019). Furthermore, RH (Huang et al., 2018; Pajunoja et al., 2014; Song et al., 2015, 2016; Zhang et al., 2015, 2018a), temperature (Maclean et al., 2017), degree of oligomerization and mass loading (Grayson et al., 2016) also impact particle viscosity. Higher RHs may result in more water to partition into the particle and act as a plasticizer which decreases its viscosity (Song et al., 2015, 2016, 2018; Zhang et al., 2011). Higher temperatures also increase the diffusion coefficient (Chenyakin, 2015). The degree of oligomerization increases the viscosity of SOA and therefore reduces its *D*_org_ as well (Grayson et al., 2017). The reduced transport of semi- or low-volatile gas species such as IEPOX onto particles also highlights the effects of phase separation on aerosol formation by decreasing reactive uptake of IEPOX (Gaston et al., 2014). This is due to increased resistance to diffusion of IEPOX through the SOA coating (Y. Zhang et al., 2018). The experimental data provided from previous studies highlight the urgency of incorporating those results into regional and global models to accurately predict the effects of phase states on aerosol formation in the ambient environment. A recent study by Schmedding et al. (2019) used a dimensionless (0-D) box model for phase-separated SOA formation at the Look Rock site during the 2013 Southern Oxidant and Aerosol Study (SOAS). Our prior work found that the inclusion of a phase-separation parameter could either inhibit SOA due to diffusion limitations in the separated organic phase or increase it by concentrating the electrolytes into the aqueous core, leading to faster acid-catalyzed reactions. This resulted in decreasing normalized mean error (NME) of the model from 83.4 % to 77.9 % and the normalized mean bias (NMB) from −66.2 % to −36.3 % compared to a previous work simulating the same dataset that assumed homogeneous aerosol (Budisulistiorini et al., 2016). The aforementioned model study (Schmedding et al., 2019) highlighted the significant impact of an organic coating layer on IEPOX-derived SOA formation but lacked any quantification of conditions that result in phase separation creating such organic coating and its phase state.

The inclusion of an explicit reaction pathway for the reactive uptake of acid-catalyzed IEPOX-derived SOA in both regional- and global-scale chemical transport models (CTMs), such as the Community Multi-Scale Air Quality Model (CMAQ v5.2.1) and the Goddard Earth Observing System (GEOS-Chem v11-02-rc), has substantially improved the performance of predicted SOA yields (Marais et al., 2016; Pye et al., 2013, 2017). These models do not include parameters in their aerosol algorithms that account for aerosol morphology or phase separation and its impact on SOA formation (Marais et al., 2016; Pye et al., 2017, 2018), which can lead to potential deviations of aerosol quantification. This work systematically examines formation of coatings comprised of OA derived from a mixture of biogenic and anthropogenic compounds. Besides predicting frequencies of core–shell morphology, this work explores how organic coating impacts acid-catalyzed multiphase reactions of IEPOX by implementing parameterizations to determine the viscosity and phase state of particles (liquid or glassy) in CMAQ and simulating for the continental United States.

## 2 Methods

### 2.1 Phase state and its impact on reactive uptake: overview

Particles that are in a liquid-like state may either be an internal homogeneous mixture, or they can be phase separated in a core–shell morphology with inorganic-rich core and the organic-rich shell also referred to as liquid–liquid phase separation (LLPS). The occurrence of LLPS depends on the average O : C ratio, organic mass to sulfate ratio, ambient temperature and ambient RH (Song et al., 2018; Zuend and Seinfeld, 2012). Organic constituents of an aerosol may also exhibit a solid-like glassy phase state when the ambient temperature is below the glass transition temperature (*T*_g_), which is a function of RH and the aerosol composition (DeRieux et al., 2018). A liquid phase state occurs when the *T*_g_ is lower than ambient temperature. The difference in viscosity (*η*_org_) of the organic-rich coating, below and above the *T*_g_, may be as high as 8 orders of magnitude (Marsh et al., 2018). Thus, *T*_g_ can be used to determine when aerosols are in a highly viscous glassy state (*η*_org_ ≥ 10^12^ Pa s), a semi-solid state (100 ≤ *η*_org_ < 10^12^ Pa s) or a liquid state (*η*_org_ < 100 Pa s) (Marcolli et al., 2004; Martin, 2000). Aerosols in a highly viscous or a semi-solid state can be homogeneous or phase separated in a core–shell morphology, similar to particles with a liquid-like state. For this study, we also ran a sensitivity simulation to see the impact if highly viscous particles were phase separated at all times (refer to Sect. 2.6). The need for this sensitivity is based on recent observations showing higher-than-anticipated rebound fractions in OA particles with viscosities > 10^2^ Pa s, implying a highly viscous particle that can likely exhibit diffusive limitations in reactive uptake (Reid et al., 2018). These viscous aerosols can be assumed to be in an amorphous solid phase, homogeneous or phase separated, but unlike liquid particles, they can only dissipate energy by rebounding, and criteria governing phase separation in them is not well constrained (Bateman et al., 2015a, b, 2017; Reid et al., 2018; Virtanen et al., 2010). The specific conditions under which a particle will form a glassy rather than liquid-like organic shell are unclear but thought to be driven by the same underlying physical properties that drive viscosity. This led to a sensitivity simulation with the consideration that semi-solid or glassy particles would inherently adopt a core–shell morphology. This sensitivity case can be thought of as an upper bound on the frequency of particles separating into core–shell morphology. For the primary phase-separation criteria to be broader, it was not assumed that a semi-solid state is always phase separated, and instead the LLPS criteria were applied for conditions that produce a low aerosol water content (refer to Sect. 2.3).

Phase state of an organic shell impacts reactive uptake by affecting the diffusivity of a species through this outer organic shell (*D*_org_). *D*_org_ can be related to the viscosity of the organic shell (*η*_org_) using the Stokes–Einstein equation, as shown in Eq. (1) (Ullmann et al., 2019):

(1) $$D_{\text{org}}=\frac{k_{\text{b}}T}{6\pi \eta_{\text{org}}r_{\mathrm{diffusive}}},$$

where *k*_b_ is the Boltzmann constant, *T* is the ambient temperature, *η*_org_ is the organic shell viscosity, and *r*_diffusive_ is the hydrodynamic radius of the molecule diffusing through the viscous organic shell.

### 2.2 Determining the glass transition temperature (*T*_g,org_)

The combined *T*_g,org_ for anthropogenic species, biogenic species and aerosol water associated with them was found using a modified version of the Gordon—Taylor mixing rule, as represented by Eq. (2) (DeRieux et al., 2018; Gordon and Taylor, 1952):

(2) $$T_{\text{g},\text{org}}=\frac{\left(w_{\text{s}}T_{\text{g},\text{w}}+\frac{1}{k_{\text{GT}}}\left(w_{\text{a}}T_{\text{g},\text{a}}+w_{\text{b}}T_{\text{g},\text{b}}\right)\right)}{w_{\text{s}}\left(\mathrm{RH}\right)+\frac{1}{k_{\mathrm{GT}}}\left(w_{\text{a}}+w_{\text{b}}\right)},$$

where *T*_g,w_ is the glass transition temperature of water (137 K) (Koop et al., 2011). *T*_g,a_ and *T*_g,b_ are the respective glass transition temperatures (K) for the anthropogenic (also includes all combustion-generated POA in addition to VOC-derived SOA; see Table 1) and biogenic (only includes VOC-derived SOA; see Table 1) fractions of OA. *K*_GT_ is the Gordon—Taylor constant, which is assumed to be 2.5 based on Koop et al. (2011). *w*_a_ and *w*_b_ are the mass fractions of anthropogenic and biogenic OA species, respectively. *w*_s_(RH) or simply *w*_s_ is the mass fraction of organic aerosol water.

For this work, it was assumed that 10 % of the aerosol water was present in the organic shell, which is a lower bound estimate of the range of organic water reported by Pye et al. (2017). Approximately 10% of total aerosol water is associated with the organic phase during daytime when IEPOX chemistry is more prevalent as indicated by the observations collected during the 2013 SOAS campaign (Guo et al., 2015). To best replicate daytime IEPOX chemistry, the 10 % value was chosen under the assumption that the underprediction of nighttime organic water would negligibly impact overall IEPOX-derived SOA. Naturally, this is not applicable for the rest of multiphase chemistry and should be addressed accordingly in future work. In CMAQ v5.2.1, the total aerosol water is predicted by ISORROPIA and only associated with inorganic electrolytes such as ammonium bisulfate (Pye et al., 2017). As represented by Eq. (3), the *w*_s_ along with the *w*_a_ and *w*_b_ make up the organic water component of the aerosol and add up to 1:

(3) $$w_{\text{s}}=1-\left(w_{\text{a}}+w_{\text{b}}\right).$$

Shiraiwa et al. (2017) used 179 organic species to fit a relationship between *T*_g_, the molar mass (*M*) and O : C ratio (Shiraiwa et al., 2017) . Following the same relationship as in Eq. (4), the respective glass transition temperatures for the anthropogenic and biogenic fractions (*T*_g,a_ and *T*_g,b_) were calculated using the weighted average molar mass (*M_x_*) and O : C ratio ((O : C)*_x_*) for all individual anthropogenic and biogenic species addressed in CMAQ (see Table 1), where *x* refers to anthropogenic (a) or biogenic (b) OA and *i* refers to individual species:

(4) $$T_{\text{g},x}\;\mathrm{or}\;T_{x}=-21.57+1.51M_{x}-0.0017M_{x}^{2}+131.4{\left(\text{O}:\text{C}\right)}_{x}-0.25M_{x}{\left(\text{O}:\text{C}\right)}_{x},$$

Where

$$M_{x}=\sum \left(w_{\text{i},x}M_{\text{i},x}\right);{\left(\text{O}:\text{C}\right)}_{x}\;=\sum \left(w_{i,x}{\left(\text{O}:\text{C}\right)}_{i,x}\right);w_{i,x}\;=\frac{{\mathrm{mass concentration}}_{i,x}}{{\mathrm{total mass concentration}}_{x}}.$$

When the ambient temperature is below the *T*_g,org_, the viscosity of the coating (*η*_org_) is assumed to remain constant at 10^12^ Pa s. When the ambient temperature is greater than or equal to the calculated *T*_g,org_, the viscosity of the organic shell is calculated using a modified Vogel–Tamman–Fulcher equation (DeRieux et al., 2018; Fulcher, 1925; Tamman and Hesse, 1926; Vogel, 1921), as shown in Eq. (5) with experimentally fitted parameters as shown in Eqs. (6) and (7):

(5) $$\log_{10}\left(\eta_{\text{org}}\right)=-5+0.434\frac{T_{0}D}{T-T_{0}}$$

(6) $$T_{0}=\frac{39.17T_{\text{g},\text{org}}}{D+39.17}$$

(7) $$D=14.4-2.3{\left(\text{O}:\text{C}\right)}_{\mathrm{avg}}.$$

*T* is the ambient temperature (K), *T*_0_ is an experimentally fitted parameter of Eq. (5) that varies as a function of *T*_g,org_ and the fragility parameter *D*, which is a function of the O : C ratio (DeRieux et al., 2018; Zhang et al., 2019). (O : C)_avg_ refers to the overall OA (including both POA – all anthropogenic and SOA – anthropogenic and biogenic; see Table 1) O : C ratio given by CMAQ.

The effective diffusion coefficient for IEPOX through the organic coating (*D*_org_) was then calculated using the Stokes–Einstein equation (refer to Eq. 1), assuming that *r*_diffusive_ = 1 nm (Evoy et al., 2019; Ullmann et al., 2019).

### 2.3 Phase separation

Unlike the case of liquid particles, phase-separation frequencies in solid or semi-solid particles are not well understood as stated in Sect. 2.1. We predict LLPS to occur for aerosols with *η*_org_ ≤ 100 Pa s or *T*_g,org_ : *T* < 0.8 (Shiraiwa et al., 2017) and when RH ≤ separation relative humidity (SRH_LLPS_) (Bertram et al., 2011; You et al., 2014). Song et al. (2018) suggests that LLPS always happens when (O : C)_avg_ ≥ 0.56, which we implemented to predict phase separation. When (O : C)_avg_ > 0.56, phase separation (or rather LLPS) is predicted based on the conditions specified in Eqs. (8) and (9). As a model simplification, solid or semi-solid phase-separated particles (SSPSs) occur following the aforementioned LLPS criteria, but when *η*_org_ > 100 Pa s or *T*_g,org_ : *T* ≥ 0.8, to create a broader scenario referred to as PhaseSep2 (see Table 3). The SRH_LLPS_ is dependent on OA composition, as shown in Eqs. (8) and (9) based on Bertram et al. (2011), Zuend and Seinfeld (2012) and Song et al. (2018):

(8) $${\mathrm{SRH}}_{\mathrm{LLPS}}=35.5+339.9{\left(\text{O}:\text{C}\right)}_{\mathrm{avg}}-471.8{\left(\text{O}:\text{C}\right)}_{\mathrm{avg}}^{2},$$

when 0.56 < (O : C)_avg_ ≤ 0.73 and 0.1 < (OM : inorganic sulfate) ≤ 15

(9) $${\mathrm{SRH}}_{\mathrm{LLPS}}=0,$$

when (O : C)_avg_ > 0.73 and 0.1 < (OM : inorganic sulfate) ≤ 15.

### 2.4 Model description and implementation

All simulations were completed in CMAQ v5.2.1 for the SOAS campaign from 1 June to 15 July 2013, with 10 d of spin-up time starting on 21 May 2013. Model inputs are described in Xu et al. (2018). The horizontal resolution of the simulation was 12km × 12km. Model vertical extent between the surface and 50 hPa (representing possible stratospheric influences) consisted of 35 layers of variable thickness. The Advanced Research Weather Research and Forecasting model (ARW) version 3.8 with lightning assimilation was used to generate the meteorological inputs for the simulations (Appel et al., 2017; Heath et al., 2016). The National Emission Inventory (NEI) 2011 v2 produced by the EPA was used to generate anthropogenic emissions. Biogenic emissions were determined using the Biogenic Emission Inventory System (BEIS) v3.6.1 (Bash et al., 2016). BEIS predicts lower emissions amounts for isoprene than the Model of Emissions of Gases and Aerosols from Nature (MEGAN) (Carlton and Baker, 2011). Therefore, emissions of isoprene were increased in this work to 1.5 times their original levels based on Pye et al. (2017), who found that this increase led to better agreement with field measurements of isoprene and OH at the Centreville site during the 2013 SOAS. Carbon Bond v6.3 (CB6r3) was used for the gas-phase chemistry in the model (Emery et al., 2015; Ruiz and Yarwood, 2013; Yarwood et al., 2010).

### 2.5 Reactive uptake

IEPOX-derived SOA is modeled with a first-order heterogeneous uptake reaction that includes a new term that accounts for diffusion limitations due to an organic coating when the aerosol phase state demands it, as described below in Eqs. (10)–(13) (Anttila et al., 2006; Gaston et al., 2014; Ryder et al., 2014; Budisulistiorini et al., 2017). The impact of organic coating was not considered in the original IEPOX reactive uptake algorithm in CMAQ (Pye et al., 2013):

(10) $${\mathrm{IEPOX}}_{\left(\text{g}\right)}\to {\mathrm{IEPOXSOA}}_{\left(\mathrm{aerosol}\right)}.$$

This first-order heterogeneous-reaction rate constant (*k*_het_) is defined as

(11) $$k_{\mathrm{het}}=\frac{SA}{\frac{r_{\text{p}}}{D_{\text{g}}}+\frac{4}{v_{\gamma }}},$$

where SA is the aerosol surface area (μm^2^m^−3^), ν is the mean molecular speed (ms^−1^) of gas-phase IEPOX estimated by Eq. (12):

(12) $$v=\sqrt{\frac{8\mathrm{RT}}{\pi {\mathrm{MW}}_{\mathrm{IEPOX}}}.}$$

*r*_p_ is the effective molecular particle radius including both the inorganic core and organic shell (m), *D*_g_ is IEPOX diffusivity in the gas phase ($1.9\cdot {\left(\text{M}{\text{W}}_{\mathrm{IEPOX}}\right)}^{-\frac{2}{3}}{\text{m}}^{2}{\text{s}}^{-1}$), MW_IEPOX_ = 118 g mol^−1^ is the molecular weight of IEPOX and *γ* is the reactive uptake coefficient:

(13) $$\frac{1}{\gamma }=\frac{1}{\alpha }+\frac{vr_{\text{p}}^{2}}{4H_{\mathrm{inorg}}RTD_{\text{a}}r_{\mathrm{core}}}\frac{1}{q\coth\left(q\right)-\frac{1}{q}}+\frac{vl_{\text{org}}r_{\text{p}}}{4H_{\text{org}}RTD_{\text{org},\mathrm{eff}}r_{\mathrm{core}}}.$$

*α* is the accommodation coefficient (0.02). *H*_inorg_ is Henry’s law coefficient into the inorganic core (3 × 10^7^ Matm^−1^). *R* is the gas constant (0.08206 Latm^−1^K^−1^mol^−1^), and *T* is the ambient temperature (K).

*D*_a_ is the IEPOX diffusivity in the aerosol core (10^−9^ m^2^ s^−1^) and *q* is the diffuso-reactive parameter as defined in Eq. (14):

(14) $$q=r_{\text{p}}\sqrt{\frac{k_{\mathrm{particle}}}{D_{\text{a}}}}.$$

*k*_particle_ is the pseudo-first-order rate constant (s^−1^) defined in Eq. (15) (Pye et al., 2013), with parameters defined in Table 2:

(15) $$k_{\mathrm{particle}}=\sum_{i=1}^{N}\sum_{j=1}^{M}k_{i,j}\left[{\mathrm{nuc}}_{i}\right]\left[{\mathrm{acid}}_{j}\right].$$

*D*_org,eff_ (m^2^ s^−1^) is the effective diffusivity of IEPOX through an organic coating compromised of the species given in Table 1 and 10 % of the total aerosol liquid water.

The contribution of organic species to the volume of the core is assumed negligible and water moves freely between the inorganic core and the organic shell, leading to approximately 90 % aerosol water in inorganic core and 10 % in the organic shell for this work as described by Pye et al. (2017). An extension of this assumption is that the inorganic ion species are concentrated entirely within the aqueous core when calculating *k*_particle_. *H*_org_(2 × 10^5^ Matm^−1^) is the effective Henry’s law constant for the organic coating and *l*_org_ is the organic shell thickness given by Eq. (16) calculated at each time step based on Riemer et al. (2009). *r*_p_ is the surface-area-weighted median particle radius based on surface area distribution of different species and *β* is the ratio of inorganic particle volume (90 % of the particle water and inorganic species) to the total particle volume (all organic species, water and inorganic species):

(16) $$l_{\text{org}}=r_{\text{p}}\left(1-\beta^{\frac{1}{3}}\right),$$

*r*_p_ is the effective aerosol radius (*m*), the same as in Eqs. (11), (13) and (14), and *r*_core_ is the aerosol inorganic core radius (*m*). *r*_core_ is defined based on Riemer et al. (2009) below:

(17) $$r_{\mathrm{core}}=r_{\text{p}}\beta^{\frac{1}{3}}.$$

Particles that did not have LLPS or SSPS morphology were assumed to form a homogeneous mixture of organics and inorganics (i.e., *l*_org_ = 0), reducing Eq. (13) to the standard CMAQ treatment.

### 2.6 Sensitivity simulations

Three sensitivity simulations were performed, besides the phase state and primary phase-separation prediction mechanism in CMAQ as detailed in Sect. 2.1–2.5 (PhaseSep2; see Table 3). A sensitivity simulation (Emissions Reduction) was conducted using the EPA’s emission reductions estimates for the year 2025 of 34 % and 48 % for NO*_x_* and SO_2_, respectively (Marais et al., 2016; Eyth et al., 2014). A second sensitivity (HighHorg) was conducted that used the same upper bound of *H*_org_ as reported by Schmedding et al. (2019) increasing the value from 2 × 10^5^ to 3 × 10^8^ Matm^−1^. To better understand the effects of viscosity on particle morphology and phase separation, a third sensitivity simulation (PhaseSep) was conducted. In PhaseSep, all particles with *η*_org_ > 100 Pa s were assumed to be automatically phase separated with a semi-solid outer core, also referred to as SSPS morphology. While guidelines laid down in Sect. 2.3 (Eqs. 8 and 9 along with phase separation always happening at (O : C)_avg_ ≤ 0.56) are now applicable only for LLPS in PhaseSep. Table 3 gives a brief description of the Non-PhaseSep simulation (base CMAQ without phase state and organic coating impacts), the updated PhaseSep2 proposed in this work and the three sensitivity simulations: Emissions Reduction, HighHorg and PhaseSep.

### 2.7 Measurement comparisons

Field data were collected using a high-resolution time-of-flight chemical ion mass spectrometer (HR-ToF-CIMS) coupled with a filter inlet for gases and aerosols (FIGAERO) and a two-dimensional gas chromatography time-of-flight mass spectrometer (GC × GC-ToF-MS) at the Centreville, AL, site during the 2013 SOAS campaign (H. Zhang et al., 2018). The combined measurements provide comprehensive and quantitative characterization of particle-phase OA composition with over 800 OA components in these data identified as SOA produced predominantly through VOC oxidation, with a time resolution of 4 h. Chemical formulas were assigned to all the species based on high-resolution peak fitting, and hence their O : C ratios and molecular weights were known, which were then used to empirically calculate the average *T*_g,org_ of the OA at the site for the duration of the entire SOAS campaign (1 June–15 July 2013). The speciated OA was estimated to account for 74 % of total fine OA mass during SOAS. The uncharacterized fraction of fine OA (organosul-fur compounds, highly oxidized multifunctional molecules (HOMs), etc.) will likely have some influence on the estimated *T*_g,org_. Also note that both techniques use thermal desorption approach to analyze OA composition which was recently shown to cause thermal decomposition for certain species (Lopez-Hilfiker et al., 2016; Cui et al., 2018). Thus, some interferences in *T*_g,org_ estimation could be expected by thermal decomposition; however, at this time, it remains unclear how substantial these interferences could be due to lack of understanding of the degree of decomposition that occurs in these analytical methods. Nevertheless, to our knowledge, this is the most comprehensive molecular-level OA speciation dataset and thus is appropriate to use for comparison with modeled *T*_g,org_ in this work.

We also use observed O : C ratios of various HOMs as reported by Massoli et al. (2018) recorded at Centreville forest site, Alabama, during the 2013 SOAS study to compare with the simulations. Massoli et al. (2018) used a high-resolution time-of-flight chemical ionization mass spectrometer with nitrate reagent ion (${\mathrm{NO}}_{3}^{-}$ CIMS) for these observations. More details are provided in the results section.

Model simulation results (PhaseSep2, HighHorg, Emissions Reduction and PhaseSep) were compared to recorded values for PM_2.5_ organic carbon mass concentration at monitoring stations that are a part of the South Eastern Aerosol Research Characterization study (SEARCH) (Hansen et al., 2003) to better constrain the parameters used in the calculation of *γ*_IEPOX_.

## 3 Results

### 3.1 Predicted aerosol phase state and phase-separation criteria

#### 3.1.1 Predicted phase state at surface

The ratio of *T*_g,org_ to the ambient *T* is the strongest indicator of the phase state of the aerosol. The mean, maximum and minimum values for *T*_g,org_ for all grid cells on the surface level and for all time steps were similar for both PhaseSep2 and PhaseSep estimated to be around 207–209, 284–289 and 137 K, respectively. This suggests that particles would be mostly semi-solid or liquid-like because of the similarity to the ambient temperature. The values of *T*_g,b_ and *T*_g,a_ were also the same for both PhaseSep2 and PhaseSep, and ranged from 160 to 301 and 230 to 311 K, respectively. This indicates that anthropogenic species have a narrower range of glass transition temperatures but overall higher values than biogenic species; however, the maximum values of *T*_g,b_ and *T*_g,a_ are more similar than their minimum values. This is attributed to the abundant biogenic acid-catalyzed IEPOx-derived SOA species, such as organosulfates and 2-methyltetrols, having a high *T*_g_ of 301 K and a viscosity of 10^12^ Pa s as shown in Table 1.

For the simulation period, the diurnal variability (i.e., between day and night) in the ambient *T* at any site was ~ 10 K, while *T*_g,org_ varied by as much as ~ 75 K within a 24 h period. This indicates that changes in the *T*_g,org_ : *T* ratio (i.e., phase state) were driven by *T*_g,org_ (i.e., composition of the organics and aerosol water in OA) rather than *T*.

#### 3.1.2 Predicted phase state: vertical distribution

**Figure 1.**
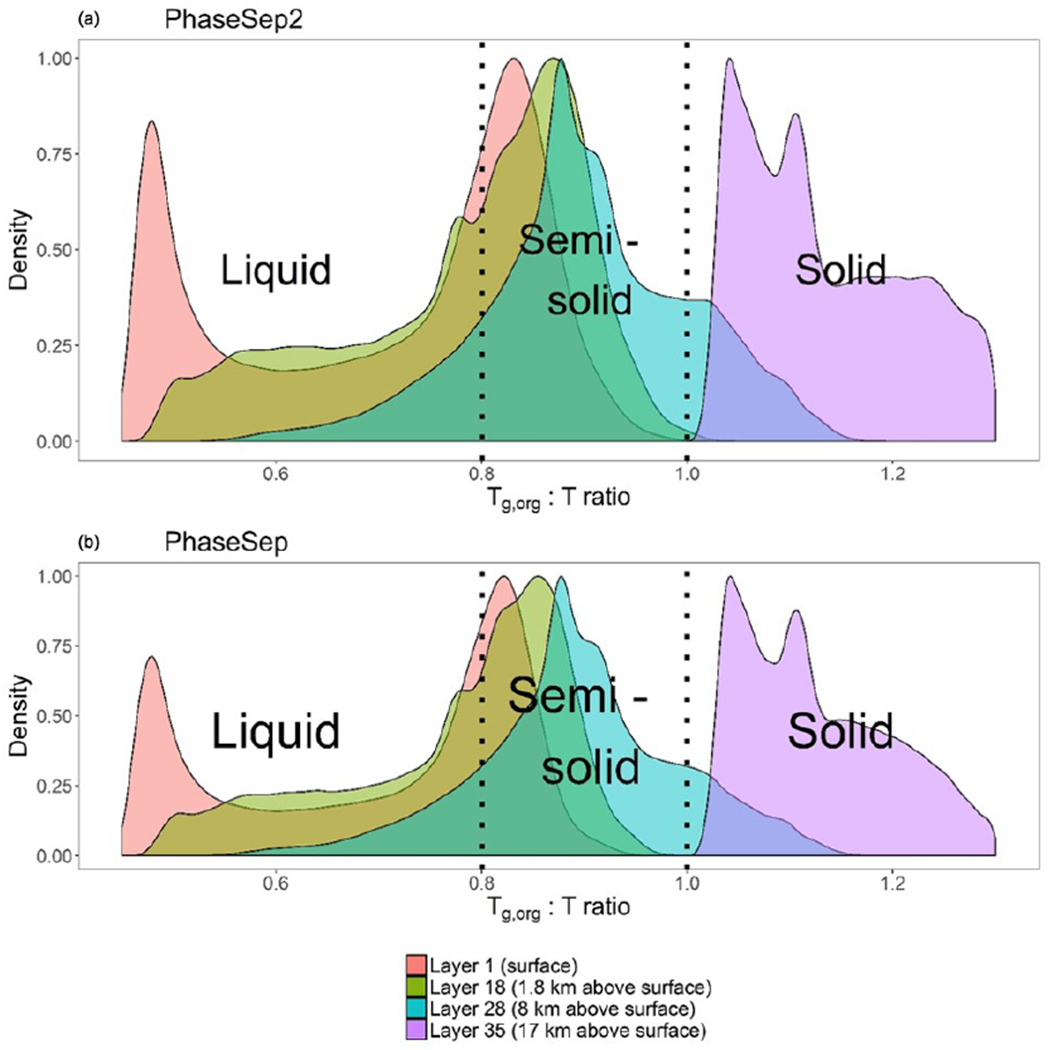
Probability density distribution of glass transition temperature to ambient temperature ratio (*T*_g,org_ : *T*) for all grid cells and time steps for **(a)** the PhaseSep2 simulation and **(b)** the PhaseSep simulation at the surface layer (red), lower troposphere (green), upper troposphere (blue) and stratosphere (purple).

Figure 1 gives the predicted probability density distribution of the *T*_g,org_ : *T* ratio for both PhaseSep2 and PhaseSep cases across all grid cells and time steps at different vertical layers of atmosphere: surface, 18th layer (lower troposphere ~ 1.8 km above ground level), 28th layer (upper troposphere ~ 8 km above ground level) and the 35th layer (lower stratosphere ~ 17 km above ground level). At the surface, the PhaseSep simulation has a minimum *T*_g,org_ : *T* ratio of 0.46 and a maximum of 0.99, while the corresponding values of the PhaseSep2 simulation were 0.48 and 0.89, respectively. In the surface layer, over 63.5 % of the *T*_g,org_ : *T* ratios were less than 0.8 in PhaseSep and 67.0 % for PhaseSep2, a value which is given as the transition point from a semi-solid viscosity to a liquid-like viscosity (Shiraiwa et al., 2017), with the remainder in a solid or semi-solid phase state. PhaseSep as expected has a higher fraction of solid or semi-solid particles, also with higher viscosity than PhaseSep2. In the lower and upper troposphere, the majority of SOA in PhaseSep and PhaseSep2 had *T*_g,org_ : *T* ratios between 0.8 and 1.0, suggesting semi-solid behavior. Particles in the lower stratosphere for both simulations exhibited *T*_g,org_ : *T* ratios > 1 that suggested a glassy state.

#### 3.1.3 Predicted phase state: spatial variability

**Figure 2.**
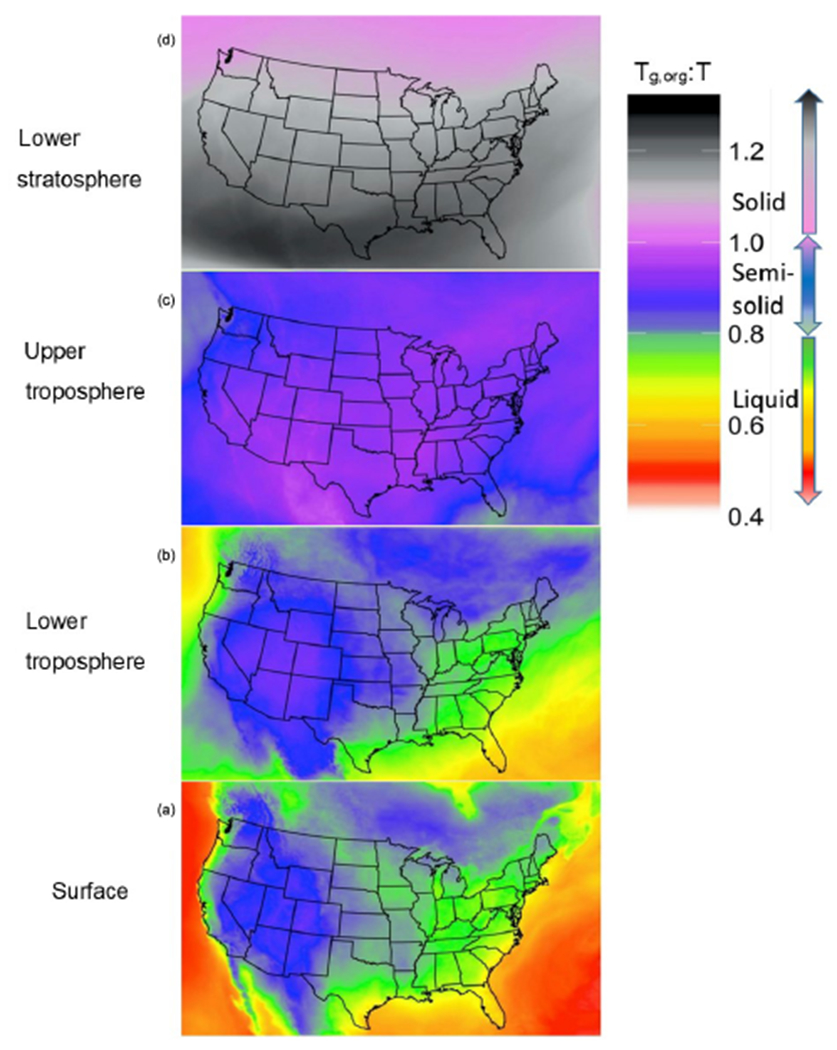
For all time steps and over the continental United States, the average glass transition temperature to ambient temperature (*T*_g,org_ : *T*) ratio at **(a)** the surface level, **(b)** 1.8 km above the surface layer (lower troposphere), **(c)** 8 km above the surface layer (upper troposphere) and **(d)** 17 km above the surface layer (stratosphere) for the PhaseSep2 simulation.

Figure 2a shows a map of the average surface layer *T*_g,org_ : *T* ratio across the continental United States for the duration of the PhaseSep2 simulation. The *T*_g,org_ : *T* ratios exhibited a bimodal distribution both at the surface and in the lower troposphere (Fig. 1), where particles over the oceans had substantially higher *w*_s_. Particles dominated by *w*_s_ had *T*_g,org_ similar to *T*_g,w_, with reduced influence from *w*_a_ and *w*_b_, which decreased their *T*_g,org_ : *T* ratio as *T*_g,w_ is substantially lower than the predicted *T*_g_ values for organic species. These particles correspond to the peak at *T*_g,org_ : *T* at over approximately 0.5 (Fig. 1). Semi-solid particles with a higher range of *T*_g_ values (Fig. 2a) were concentrated over areas associated with higher anthropogenic SOA (including anthropogenic POA listed in Table 1) and a low RH, aerosol liquid water content and biogenic SOA, such as the American southwest and Rocky Mountains. These higher *Tg* values pulled the overall *T*_g,org_ value up closer to the ambient temperature and thus brought the *T*_g,org_ : *T* ratio closer to 1, which is shown in the cluster of peaks between *T*_g,org_ : *T* = 0.8 and *T*_g,org_ : *T* = 1.

Figure 2b, c and d show the spatial profiles of the mean *T*_g,org_ : *T* ratio for each grid cell at the 18th layer of CMAQ (lower troposphere), 28th layer of CMAQ (upper troposphere) and the 35th layer of CMAQ (stratosphere). The value of *T* drops with the decreasing pressure. The O : C ratio of the particles are predicted to increase when compared to the surface due to atmospheric oxidation. The mean O : C ratio of all particles at the surface was 0.73, while across the troposphere (at layers 18 and 28) it increased slightly to 0.75, and at layer 35 it was 0.77. The PhaseSep2 simulation followed a similar pattern of increasing O : C ratios as PhaseSep but starting higher at surface ~ 0.75 and increasing to 0.79 at layer 35. Species with high O : C (>1.6) parameterized in CMAQ as anthropogenic OA collectively can be used as a surrogate for highly oxygenated organic aerosol (OOA), specifically low-volatility oxygenated organic aerosol (LVOOA) and semi-volatile oxygenated organic aerosol (SVOOA). Table 1 shows the species that led to this specific modeled change in O : C with elevation. The mean mass fraction of anthropogenic OA increased from ~ 40% at surface to ~ 65% at the upper troposphere and eventually ~ 80% at layer 35 in both PhaseSep and PhaseSep2, whereas biogenic OA composed of isoprene-derived OA drops from ~ 30% at surface to 24 % at the upper troposphere and eventually ~ 20% at layer 35. The higher O : C in PhaseSep2 relative to PhaseSep is connected to higher biogenic OA as well (Fig. 4c and e). This is in agreement with the findings from airborne measurements in the southeastern United States as part of the Southeast Nexus (SENEX) field campaign that show a sharp drop in isoprene-derived OA and drastic increase in OOA with rising altitude (Xu et al., 2016). The mean value of *T*_g,org_ also increased from 207 K at the surface to 219 K at layer 18, 223.5 K at layer 28 and 239 K at layer 35. This change in *T*_g,org_ was primarily driven by decreases in organic water in the aerosol, which decreased from an average 29 % at the surface to 1.4% at layer 35. The removal of water from the organic phase led to the disappearance of the bimodal *T*_g,org_ : *T* ratio beyond the 28th layer (upper troposphere).

The mean *T*_g,org_ : *T* ratio was less than 1 in the lower troposphere for PhaseSep and PhaseSep2. PhaseSep predicted 59.7% of particles were likely to be liquid based on the *T*_g,org_ : *T* ratio < 0.8, while PhaseSep2 predicted that 45.4% would likely be liquid-like. The remaining semi-solid particles were still concentrated over the American southwest and Rocky Mountains. The difference between *T*_g,org_ : *T* ratio was more pronounced in the upper troposphere, where PhaseSep predicted 69.4% of particles would have semi-solid behavior, with the remainder almost evenly split between liquid-like and glassy behavior. On the other hand, PhaseSep2 predicted over 99.6 % of particles in the upper troposphere would be semi-solid. At the 35th layer of CMAQ, all particles in both simulations had a *T*_g,org_ : *T* ratio > 1, indicating glassy viscosities. Particles with the highest *T*_g,org_ : *T* ratio at this altitude were located over the southern half of the simulation area, with *T*_g,org_ : *T* ratios approaching ~ 1 in the northern half of the simulation. Particles in the northern half of the continental United States domain had higher concentrations of biogenic and anthropogenic SOA in comparison to those in the southern half of the domain and therefore had higher *T*_g,org_ values than their southern counterparts had.

#### 3.1.4 Phase-separation frequency with different phase-separation criteria

Whether a particle is semi-solid or liquid, and whether it has LLPS or SSPS morphology, is influenced by the proportions of SOA constituents, including water. The overall phase-separation frequency using the PhaseSep criteria was 68.5 %, where 54.8 % of predicted viscosities were greater than 100 Pa s, indicating that they exhibited SSPS morphology. The median viscosity of SSPS particles was on the order of 10^3^ Pa s, just above the threshold where particles start exhibiting a glassy state. The remaining 13.7% of the phase-separated particles exhibited a LLPS morphology. The median viscosity of LLPS particles was on the order of 10^0^ Pa s, indicating a very liquid-like state. *D*_org,eff_, which is inversely related to *η*_org_ as derived from Eq. (1), had a mean and median of 3.40 × 10^−12^ and 3.94 × 10^−11^ m^2^s^−1^, respectively. The mean O : C ratio of LLPS particles was 0.68. The mean values of *w*_a_ and *w*_b_ in LLPS were 37.7% and 19.8%. The largest observed contributions of *w*_a_ and *w*_b_ to the total organic mass were 96.9 % and 82.1 %. This suggests that anthropogenic aerosol components are more dominant than the biogenic components, whereas biogenic components are likely more water soluble with their average O : C being 0.72 compared to 0.59 for anthropogenic constituents (Table 1). The mean fraction of the organic shell composed of water (*w*_s_) was 42.4 % and a maximum of 99.9 %.

The removal of the hypothetical assumption that all particles with a semi-solid viscosity were phase separated (PhaseSep2) decreased the overall phase-separation frequency to 29 % from 68.5 % in the PhaseSep simulation. The entirety of this reduction was from reductions in SSPS only. The phase-separation frequency at the Centreville, Alabama, site decreased to 65.4 % from 79.3 %, which is still in agreement with the values reported by Pye et al. (2017).

#### 3.1.5 Diurnal influence of OA composition on phase state

**Figure 3.**
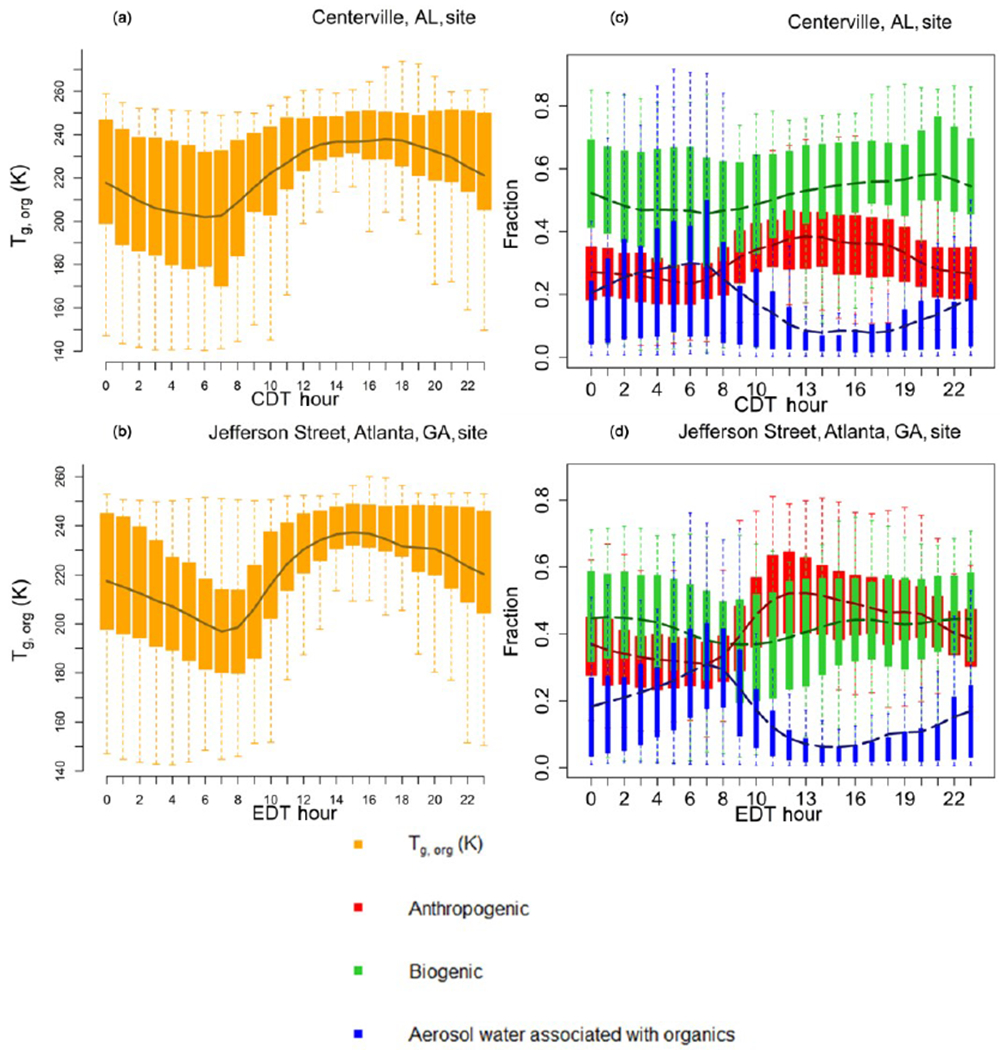
Diurnal pattern for SOAS (1 June-15 July) 2013 of the organic glass transition temperature (*T*_g,org_ – orange), and contributions of anthropogenic OA (red), biogenic OA (green) and aerosol water associated with organics (blue) to *T*_g,org_ in the PhaseSep2 case at the **(a, c)** Centreville, AL, site and the **(b, d)** Jefferson Street, Atlanta, GA, site. Bars/shaded boxes indicate 25th to 75th percentiles. Extreme bounds of whiskers indicate 5th to 95th percentiles (i.e., 95 % confidence interval), and the line indicates the mean.

Figure 3 shows the diurnal profile of model extracted *T*_g,org_ and relative contributions of the model-predicted anthropogenic, biogenic and water fractions to *T*_g,org_ for 1 June–15 July 2013 at two SEARCH monitoring sites: a rural site in Centreville, Alabama (Fig. 3a and c), and at an urban site at the Jefferson Street, Atlanta, Georgia (Fig. 3b and d). These diurnal profiles are pretty much same for both PhaseSep2 and PhaseSep cases. Both the Centreville and Atlanta sites (Fig. 3) had *T*_g,org_ values that ranged ~ 150–250 K (Fig. 3). The rural Centreville site is dominated by biogenic OA (Fig. 3c), whose diurnal trend is similar to that of overall *T*_g,org_ at this site (Fig. 3a), whereas the Jefferson Street site has a significant presence of both anthropogenic and biogenic OA, but the diurnal trend of *T*_g,org_ at this site (Fig. 3b) is similar to anthropogenic OA which dominates slightly (Fig. 3d). Figure 3 also gives the diurnal pattern of relative contribution of aerosol liquid water (associated with organics) to *T*_g,org_. The peaks in *T*_g,org_ coincided with the daytime period of high emissions of VOCs and lower contribution of aerosol liquid water (Fig. 3). At night and in the early morning, due to higher contribution of aerosol liquid water, *T*_g,org_ is lower than in daytime for both the sites (Fig. 3). *T*_g,org_ at the urban Jefferson Street site (Fig. 3b) also shows a sharper contrast between day and night compared to rural Centerville site (Fig. 3a), due to the more pronounced diurnal variations in aerosol liquid water (Fig. 3d). Both sites have a relatively high contribution of aerosol water (generally true for eastern United States; see Fig. 2a) to the organic phase, especially at night and in the early morning for 18:00–08:00 LT (Fig. 3).

#### 3.1.6 Predicted viscosity

**Figure 4.**
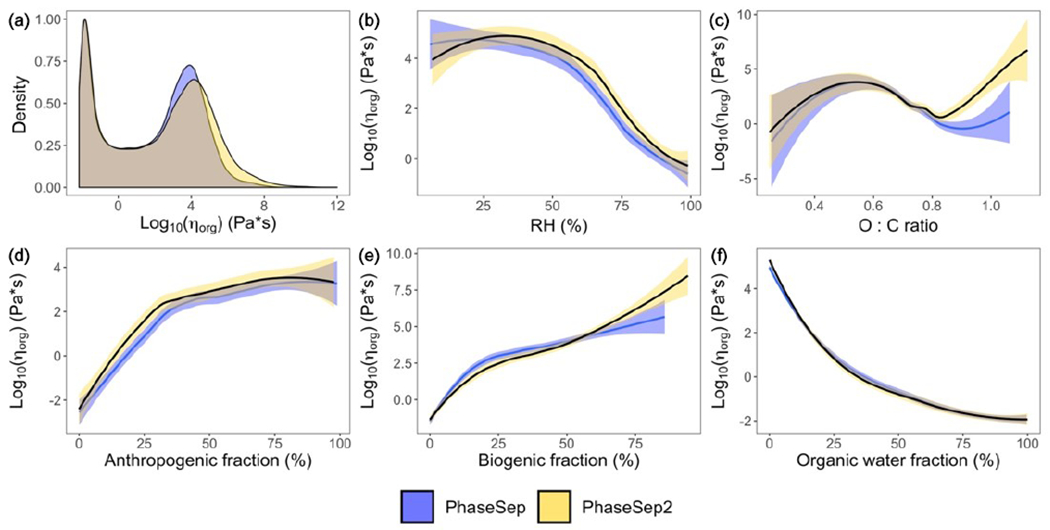
For all grid cells and time steps at the surface layer, the **(a)** probability distribution of the organic-phase viscosity and correlations of particle viscosity (*η*_org_) with **(b)** relative humidity, **(c)** atomic oxygen to carbon (O : C) ratio, **(d)** anthropogenic SOA weight fraction, **(e)** biogenic SOA weight fraction and **(f)** organic-phase water content (*w*_s_) for PhaseSep (blue) and PhaseSep2 (gold).

Figure 4a gives the probability density distribution of *η*_org_ at the surface level for all grid cells and time steps for both the PhaseSep and PhaseSep2 simulations. Both simulations predicted a bimodal distribution of *η*_org_ with comparable median values. Semi-solid and glassy particles tended to have slightly higher viscosities in PhaseSep2 comparison to comparable particles in PhaseSep. Due to large differences in the mean and median of the predicted *η*_org_ in both simulations, we chose to use the median value as a more robust measure of the central tendency of the predicted *η*_org_ for inter- and intra-simulation comparisons. Figure 4b–f show the impacts of different external and internal factors on *η*_org_ RH was more strongly correlated with *η*_org_ (*r* = 0.68) than O : C ratio (*r* = 0.62), which was very weakly correlated with viscosity (Fig. 4b–c), The abundance of water relative to organics (Fig. 4f) drives the variability in viscosity and phase separation. Figure 4d–f show that *w*_s_ (i.e., water related to organic shell) had the strongest correlation with *η*_org_ (*r* = 0.79), followed by *w*_b_ (*r* = 0.75) and *w*_a_ (*r* = 0.63).

#### 3.1.7 Comparison to observed data

**Figure 5.**
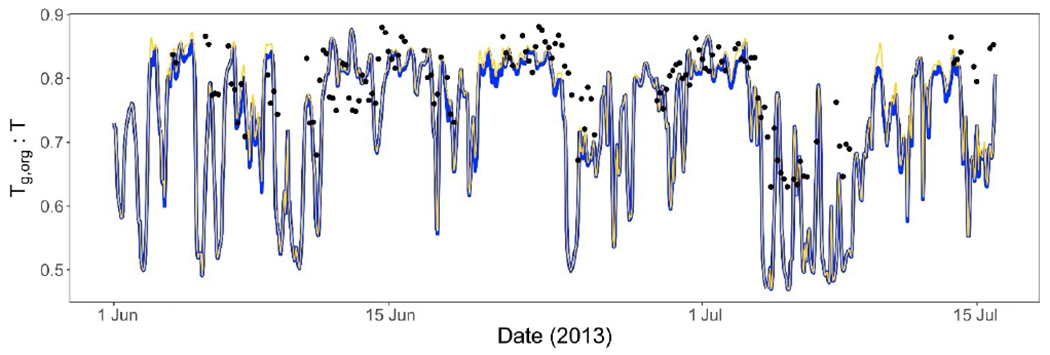
Predicted glass transition temperature to ambient temperature ratio (*T*_g,org_ : *T*) at the Centreville, AL, site during the 2013 SOAS campaign based on OA composition reported by H. Zhang et al. (2018) (black). Predicted *T*_g,org_ : *T* from this work is shown in blue for PhaseSep and gold for PhaseSep2.

Figure 5 shows the *T*_g,org_ : *T* ratio calculated from speciated organic aerosol composition field data at the Centreville, Alabama, field site at 32.944° N, 87.1386° W (H. Zhang et al., 2018), during the 2013 SOAS period. *T*_g,org_ : *T* ratio derived from data collected by H. Zhang et al. (2018) ranged from ~ 0.63 to 0.88. Meanwhile, the predicted *T*_g,org_ : *T* ratios using both PhaseSep2 and PhaseSep ranged ~ 0.47 to 0.88, slightly exceeding the range predicted from observations. It should be noted that the H. Zhang et al. (2018) observations were recorded every 4 h for ~ 60 % of the 2013 SOAS time period. Modeled *T*_g,org_ : *T* mostly captures the peaks and drops, which the field-observation-derived *T*_g,org_ : *T* shows (Fig. 5). Some mismatch can be attributed to the lack of an explicit mechanism to compute organic aerosol water uptake in CMAQ and some unaccounted SOA formation mechanisms. Further, H. Zhang et al. (2018) only accounted for ~ 70 % of SOA species listed in Table 1. The mean *T*_g,org_ : *T* ratio predicted from the 2013 SOAS Centerville site field observations was 0.79 compared quite close to the corresponding value of ~ 0.73 predicted by both PhaseSep2 and PhaseSep simulations in CMAQ. The model-estimated *T*_g,org_ : *T* (both PhaseSep2 and PhaseSep) compared to *T*_g,org_ : *T* range based on field observations at Centreville, AL, for the 2013 SOAS gives a correlation coefficient of ~ 0.64 between them. There is also a discernable consistent diurnal trend across the 2013 SOAS period for *T*_g,org_ : *T* (Fig. 5), such as stronger contribution of aerosol liquid water for 18:00—08:00 LT and a lowering of the *T*_g,org_ : *T* during those hours (Fig. 3a and c).

Massoli et al. (2018) reported the observed O : C at the Centreville, AL, site during the 2013 SOAS that ranged between ~ 0.5 and 1.4 and averaged 0.91. Massoli et al. (2018) presents the first instance of ambient measurements with a ${\mathrm{NO}}_{3}^{-}$ CIMS in an isoprene-dominated environment and identified organic nitrates or organonitrates (ONs) originating from both isoprene and monoterpene to be a significant component of the ${\mathrm{NO}}_{3}^{-}$ CIMS spectra and dominating the observed SOA at the Centreville site throughout the day, reflecting daytime and nighttime formation pathways. Both isoprene- and monoterpene-derived ONs have very high O : C (> 1) and account for up to 10 % of total oxygen at the Centreville site (Xu et al., 2015; B. H. Lee et al., 2016), explaining the high overall observed average O : C. Our modeled O : C at the Centerville, AL, site during the 2013 SOAS ranged between ~ 0.5 and 1 and averaged ~ 0.7 for both PhaseSep2 and PhaseSep. CMAQ v5.2.1 with carbon bond chemistry (used in this study) uses aero6 aerosol mechanism, without any explicit representation of formation pathways of isoprene- and monoterpene-derived ONs. Specifically CMAQ with aero6 significantly underestimates monoterpene oxidation that accounts for ~ 50 % of organic aerosol in the southeastern United States in summer (H. Zhang et al., 2018). Consideration of explicit monoterpene organic nitrates and updated monoterpene photooxidation yields in aero7 eliminates the CMAQ model-measurement bias (Xu et al., 2018). The lack of explicit organic nitrates here can explain the lack of high O : C (> 1) predictions at the Centreville, AL, site during the 2013 SOAS leading to the low correlation of model-estimated *T*_g,org_ : *T* with observations.

**Figure 6.**
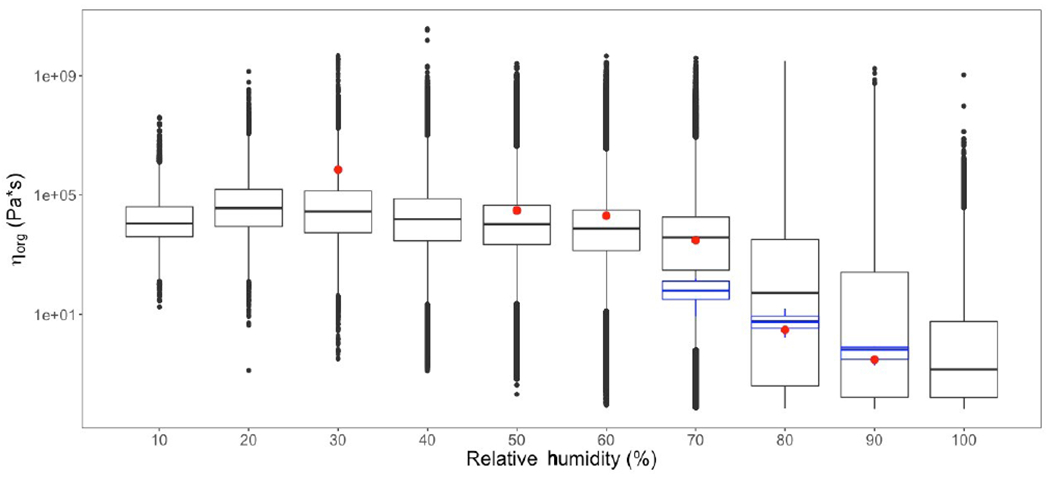
Model-predicted SOA viscosity (*η*_org_) and experimental data for *η*_org_ from Y. Zhang et al. (2018) (red) and Song et al. (2016) (blue) at varying RH.

Figure 6 shows the predicted viscosity of our phase-separation implementation for all days, grid cells and layers sorted into 10 % RH bins. The trends in range of modeled *η*_org_ are the same as those in Fig. 4b, with higher mean and quantiles of *η*_org_ corresponding to lower RH, and vice versa for higher RH. Also shown in Fig. 6 are viscosities of aerosols made in the laboratory. The red dots represent the viscosities of α-pinene SOA measured by Y. Zhang et al. (2018), and the blue box plots represent the range of viscosities of toluene SOA measured by Song et al. (2016). Both laboratory-based experimental studies show good agreement at atmospherically relevant RH ranges with the viscosities predicted by our implementation. At lower RH ranges (~ 30 %), the experimentally measured viscosities are slightly higher than those predicted by our study. This could be attributed to shattering of highly viscous SOA (*η*_org_ ≥ 10^6^ Pa s) for RH ≤ 30 % that inhibits their flow in laboratory measurements of *η*_org_ (Renbaum-Wolff et al., 2013; Zhang et al., 2015; Y. Zhang et al., 2018). Huang et al. (2018) speculates that differences in physicochemical properties of *α*-pinene SOA, including viscosity, can exhibit a “memory effect” of the conditions under which the particle formed. This is regardless of the subsequent conditions to which the particle is exposed. This could lead to differences between model-predicted and experimentally measured viscosities, as these memory effects are not well characterized. Grayson et al. (2016) reports that the viscosity of *α*-pinene SOA may vary as a function of the mass loading conditions with higher mass loading leading to lower viscosity measurements. Unfortunately, there is also a lack of experimental data on viscosity measurements at RH < 60%.

### 3.2 Impact on model predictions

#### 3.2.1 Reactive uptake coefficient of IEPOX (*γ*_IEPOX_)

**Figure 7.**
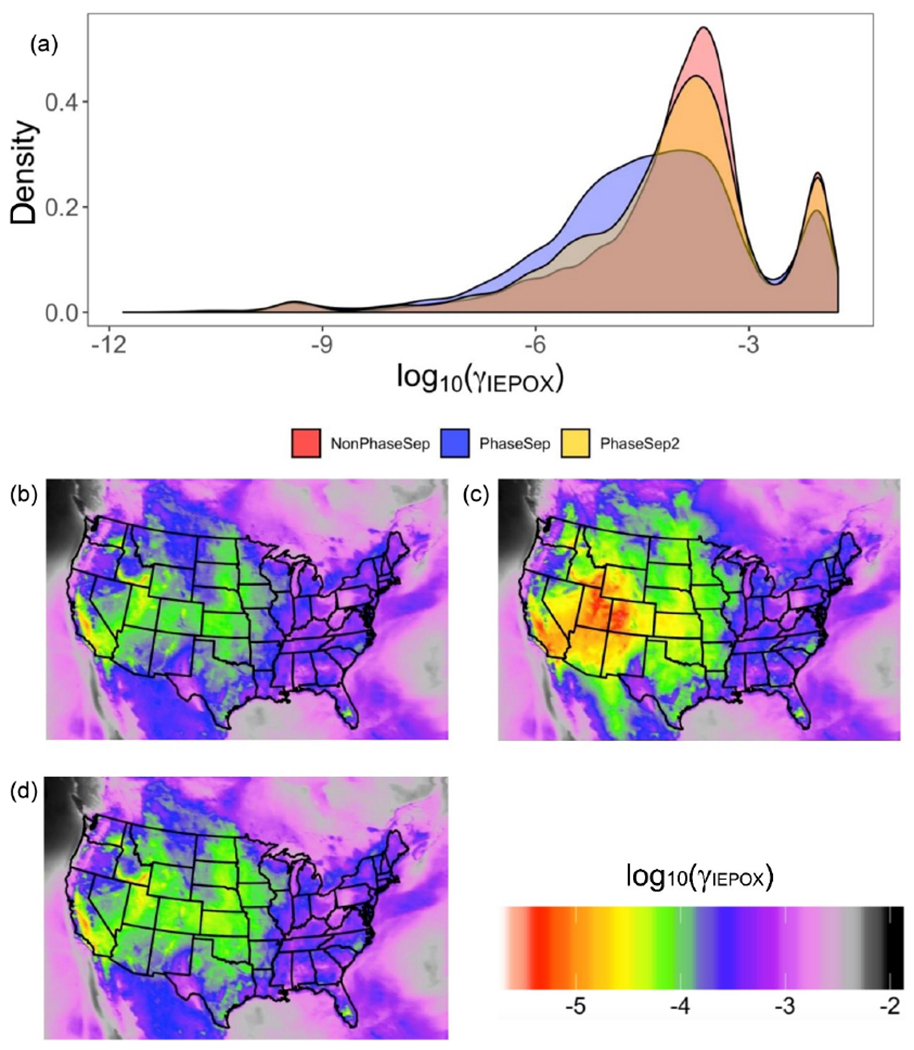
For all grid cells and time steps, the predicted **(a)** probability distribution of *γ*_IEPOX_ at the surface level for the NonPhaseSep (red), PhaseSep (blue) and PhaseSep2 (gold) simulations. For each grid cell, the mean value of *γ*_IEPOX_ for the **(b)** NonPhaseSep, **(c)** PhaseSep and **(d)** PhaseSep2 simulations.

Previous experimental studies show that phase separation forming semi-solid organic aerosol coatings is expected to decrease IEPOX reactive uptake (*γ*_IEPOX_) and thus the resulting SOA (Y. Zhang et al., 2018). Figure 7 clearly is in agreement with Y. Zhang et al. (2018), showing reductions in *γ*_IEPOX_ with PhaseSep, and PhaseSep2 simulations relative to the NonPhaseSep. Compared to the original CMAQ with no phase separation considered (NonPhaseSep), PhaseSep had a ~ 18 % decrease for mean *γ*_IEPOX_ at the surface level, while PhaseSep2 led to a reduction of only ~ 2 % (Fig. 7a). For the southeastern United States, a similar overall shift of higher *γ*_IEPOX_ values > 10^−3^ in NonPhaseSep to lower values ranging between 10^−4^ to 10^−6^ occurs with the introduction of phase separation and phase state parameters in CMAQ, much more in PhaseSep than in PhaseSep2 (Fig. S1 in the Supplement).

Across the continental United States, for different locations as well, there is a significant reduction in mean *γ*_IEPOX_ for the 2013 SOAS period in PhaseSep compared to NonPhaseSep (Fig. 7b and c); however, it is much more similar between PhaseSep2 and NonPhaseSep (Fig. 7b and d). There was high variability in the value of *γ*_IEPOX_ between regions: specifically, between the eastern and western United States. To understand the drivers that influence changes in γ_IEPOX_> with the new PhaseSep2 and PhaseSep simulations, grid cells that exhibited the maximum increase and decrease relative to the NonPhaseSep were analyzed. When phase separation was included, particles in grid cells and time steps with the maximum reduction in *γ*_IEPOX_ were the result of a low *D*_org,eff_ of 5.83 × 10^−19^m^2^s^−1^ and an *l*_org_ as high as 100 nm, i.e., thick organic coating with diffusion limitations. Particles in the grid cell and time step with the highest increases in *γ*_IEPOX_ had a *D*_org,eff_ value of 7.34 × 10^−16^ m^2^ s^−1^ and *l*_org_ of 0.67 nm, and were located over oceans with an abundant amount of aerosol liquid water that were in close proximity to biogenic isoprene emission sources (Fig. S2). These large increases in *γ*_IEPOX_ were primarily caused by increases in *k*_particle_ due to added nucleophiles (i.e., abundant aerosol liquid water) and a lack of diffusive limitations through the organic shell. Regions with highest reductions in mean *γ*_IEPOX_ for the 2013 SOAS period across the continental United States in PhaseSep (southwestern US and southern Canada; Fig. 7c) and PhaseSep2 (midwestern US; Fig. 7d) relative to NonPhaseSep (Fig. 7b) also had higher *l*_org_ (Fig. S2). To summarize, the phase (which influences *D*_org,eff_) and thickness (*l*_org_) of the organic coating are the main drivers of change in *γ*_IEPOX_.

A recent study by Riva et al. (2019) demonstrated that the formation of organosulfates during the IEPOX reactive uptake process leads to an organic coating and thus a reduced *γ*_IEPOX_. This manifests as a self-limiting effect during the IEPOX-derived SOA formation. Atmospheric models, including this work, do not consider this recently observed self-limiting process yet, but accounting for it may lead to a further reduction of the *γ*_IEPOX_. It is also interesting to note that the spatial maximum of mean organic coating thickness across the continental United States for PhaseSep2 and PhaseSep cases came around the thin (~ 20 nm) and thick (~ 40 nm) organic coating as used in Riva et al. (2019), respectively (Fig. S2).

#### 3.2.2 Predicted SOA mass

**Figure 8.**
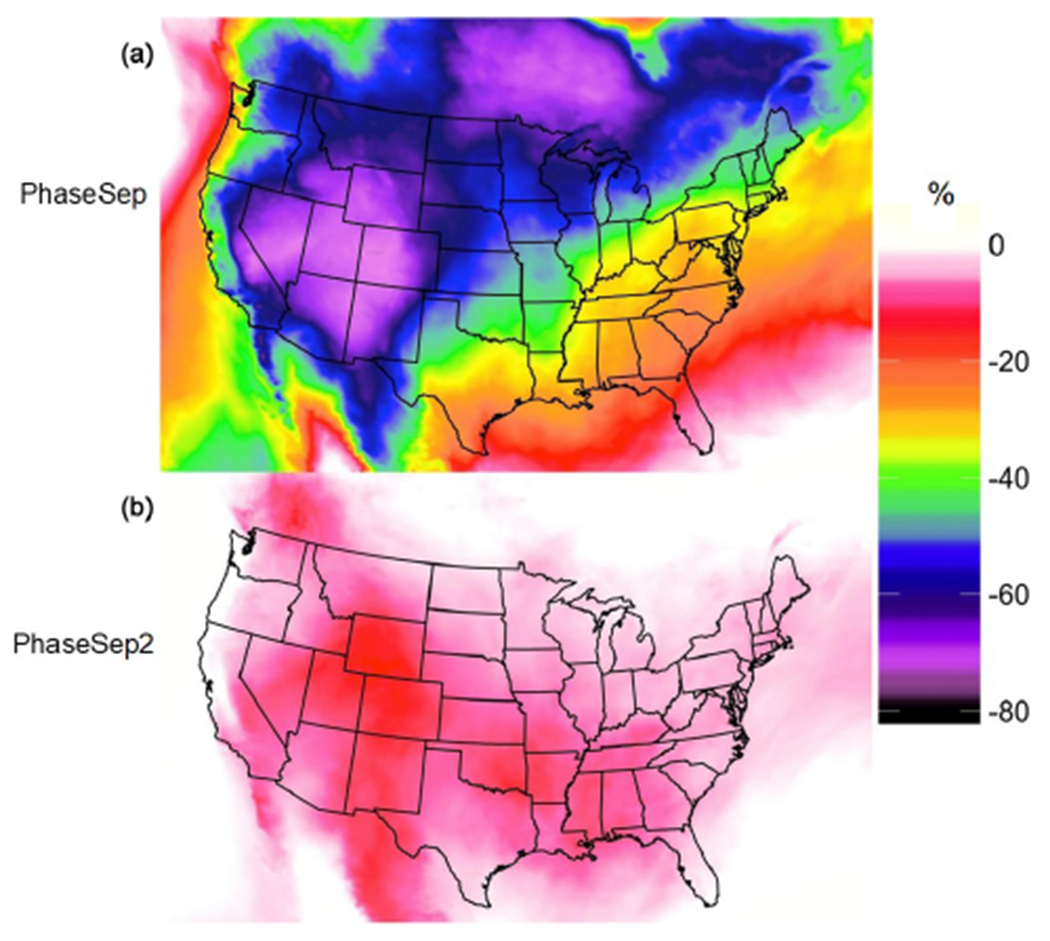
Spatial map of the mean percent relative change in IEPOX-derived SOA for the **(a)** PhaseSep and **(b)** PhaseSep2 cases relative to the NonPhaseSep simulation.

Variability in *γ*_IEPOX_, in the PhaseSep2 and PhaseSep relative to the NonPhaseSep (Fig. 7), was also reflected in the large geospatial variations in the concentrations of IEPOX-derived SOA, i.e., organosulfates and tetrols (Fig. 8). Higher reductions in the IEPOX-derived SOA for PhaseSep relative to NonPhaseSep were also in regions such as the southwestern United States and southern Canada (more pronounced near the Great Lakes), with higher reductions in *γ*_IEPOX_ due to thick organic coatings (Fig. S2). Although the southwestern United States shows a high reduction in IEPOX-derived SOA (Fig. 8), it is not reflected for changes in biogenic SOA, while the high reduction in IEPOX-derived SOA (Fig. 8) in southern Canada is reflected in reductions in biogenic SOA in that region. This spatial variability can be explained by the lower fraction of IEPOX-derived SOA in total biogenic SOA on average in the southwestern United States compared to its higher fraction in southern Ontario (Fig. S3), which is further reduced to some extent in PhaseSep2 (Fig. S3c) and becomes negligible in the PhaseSep case for the southwestern US (Fig. S3b). Hence, the magnitude of changes in biogenic SOA and eventually PM_2.5_ organic carbon mass (Fig. S4) is dampened as compared to changes in IEPOX-derived SOA mass with introduction of phase-separation parameters (Fig. 8).

On average, the largest reduction in biogenic SOA mass at any one grid cell was 40.9% and occurred over a forested region in southern Ontario near Lake Superior which also exhibits high IEPOX-derived SOA contribution to total biogenic SOA (Fig. S3). For the southeastern United States, modeled average reductions for 2013 SOAS period in IEPOX-derived SOA ranged between 25 % and 30 %, translated to a 10 %−15 % reduction in total biogenic SOA (Fig. 8). The highest average reduction in IEPOX-derived SOA was 74.06 % over Colorado (Fig. 8a). This reduction matters less in terms of overall biogenic SOA reduction due to negligible contribution of IEPOX-derived SOA to total biogenic SOA in the American southwest (Fig. S3). In southern Ontario, where the maximum biogenic SOA reduction in PhaseSep occurred, the average reductions in IEPOX-derived SOA per grid cell ranged from 63 % to 66 %. The southern Ontario region with maximum biogenic SOA reduction had average particle viscosities per grid cell in the range of ~ 10^3^ to 10^6^ Pa s. The total phase-separation frequency of particles for southern Canada region was 86.3 % of which SSPS was 62.04% and LLPS was 24.26%. The combination of these factors led to a 52.64 % average reduction in *γ*_IEPOX_ in PhaseSep, which can be treated as a hypothetical upper bound.

PhaseSep2 led to predicted *γ*_IEPOX_ values that were more similar to those of NonPhaseSep than PhaseSep; however, there is some small variation in western states. Overall, biogenic SOA mass yields increased by an average of 25.86 % from the PhaseSep simulation for the continental United States. Table S1 in the Supplement shows a modest 4% improvement in model performance for total PM_2.5_ OC mass, in the isoprene-abundant southeastern United States with PhaseSep2. The range of phase-separation frequency in semisolid particles per grid cell is still wide in PhaseSep2, i.e., 0.02 % to 55.8 % SSPS. Increased frequency of bulk phase in semi-solid conditions in PhaseSep2 relative to PhaseSep causes much less resistance to reactive uptake, closer to but still more than in NonPhaseSep. This is reflected in the similarity of *γ*_IEPOX_ between PhaseSep2 and NonPhaseSep (Fig. 7c). Hence, a smaller difference in IEPOX SOA and biogenic SOA in PhaseSep2 relative to NonPhaseSep occurs, unlike much higher differences observed in PhaseSep (Figs. 8 and S3). Particles in PhaseSep2 adopted a core–shell morphology less frequently than those in PhaseSep, typically causing lower *k*_particle_ (Eqs. 13 and 14), which led to reduced reactive uptake of IEPOX compared to PhaseSep.

#### Comparison to observed data

**Figure 9.**
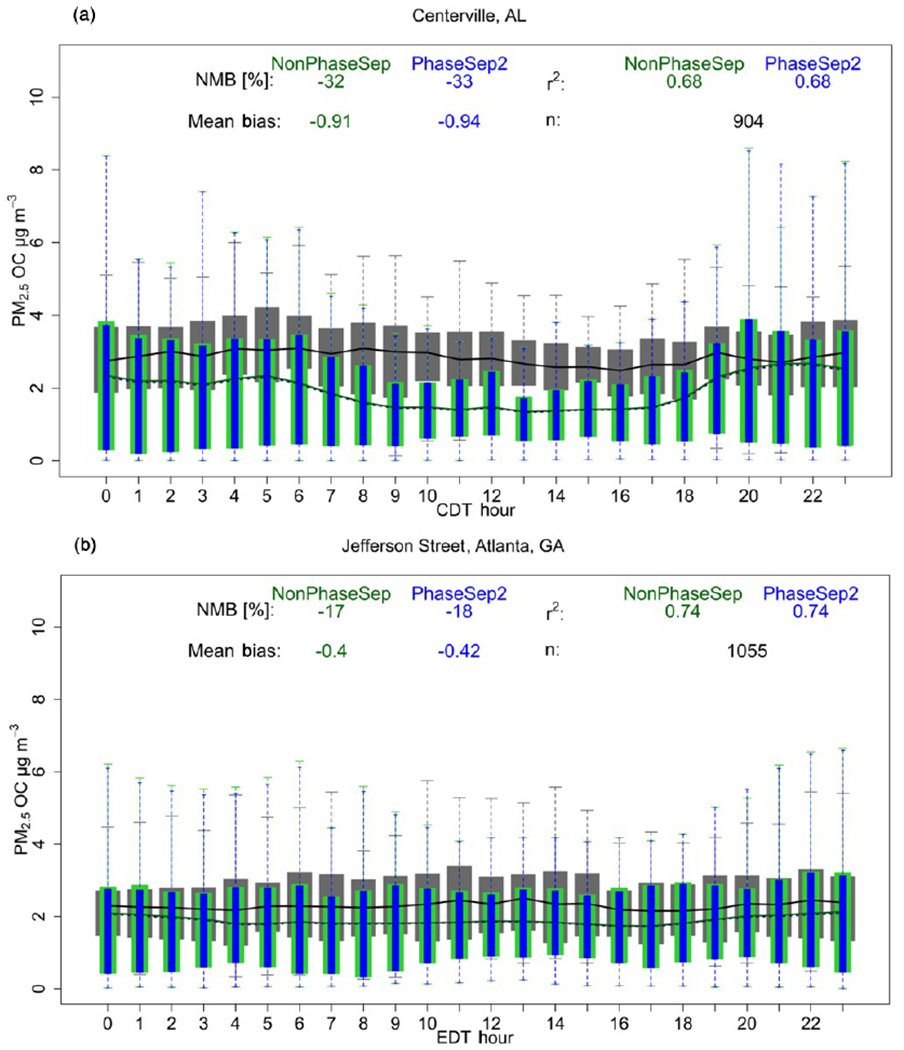
PM_2.5_ organic carbon (OC) mass (μgm^−3^) as a function of hour of the day. Non-aggregated performance statistics – mean bias (μgm^−3^), % normalized mean bias (NMB) and Spearman’s correlation coefficient (*r*^2^) of NonPhaseSep (green) and PhaseSep2 (blue) cases relative to observed (grey) PM_2.5_ OC mass for the **(a)** rural Centreville, AL, site and **(b)** urban Jefferson Street, Atlanta, GA, site. Bars/shading indicate 25th to 75th percentiles. Extreme bounds of whiskers indicate 5th to 95th percentiles (i.e., 95 % confidence interval). Lines indicate means (dashed line indicates PhaseSep2). *n* is the number of observation points.

Figure 9 and Table S1 show that different consideration of phase state and separation in CMAQ can impact model performance differently. PhaseSep slightly worsened the NMB based on comparison with hourly PM_2.5_ organic carbon mass SEARCH observations at both the Centreville rural site and urban Jefferson street, Atlanta, site by ~ −4 %. However, this change was marginal in terms of mean bias change in PhaseSep relative to the NonPhaseSep case being < 0.1 μg m^−3^. The sensitivity cases that assumed a higher *H*_org_ (HighHorg) and considered LLPS criteria in predicting SSPS (PhaseSep2) resulted in correcting the worsening of model performance observed with the PhaseSep case (Fig. 9 and Table S1). The initial assumption regarding phase separation at high viscosity seems to be approximately as important as the assumption regarding *H*_org_ in constraining the impact of phase state and morphology on reactive uptake of IEPOX. This highlights the poorly constrained parameters in models such as *H*_org_ assumed as a constant and less understood criterion that might govern phase separation under low RH or low aerosol water at different O : C ratios.

### 3.3 Sensitivities

**Figure 10.**
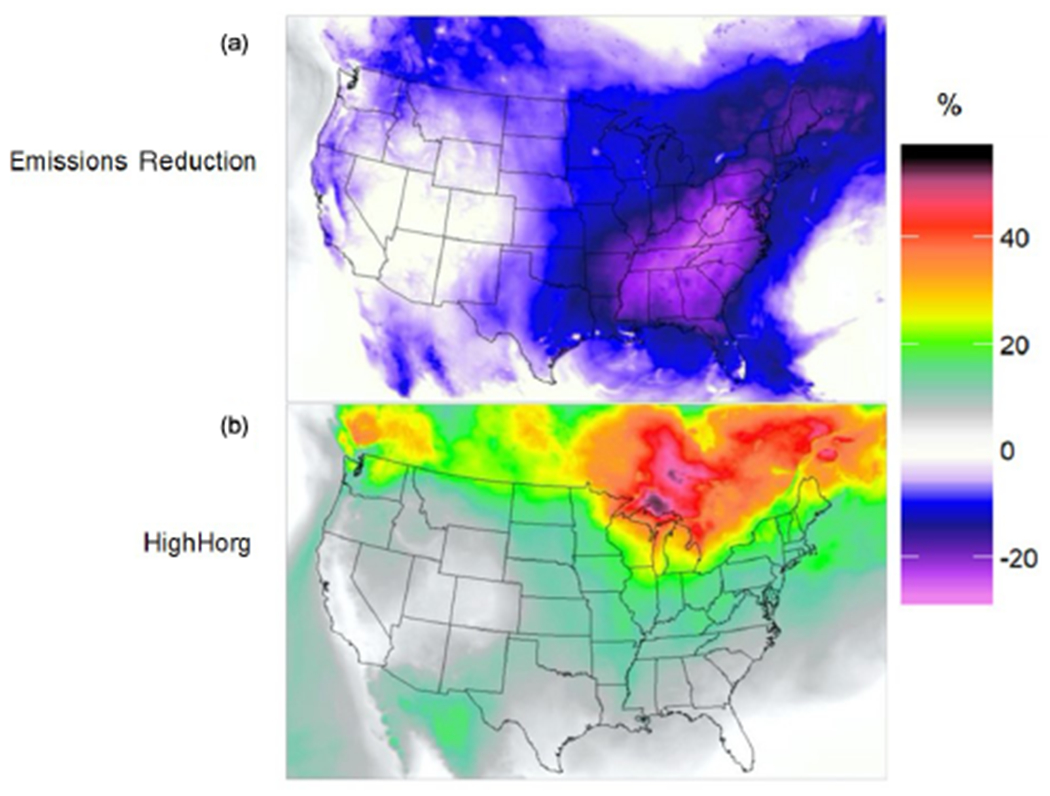
Relative change (%) in biogenic SOA mass at the surface level from the PhaseSep parameterization for the **(a)** NO*_x_* and SO_2_ Emissions Reduction sensitivity simulation and **(b)** HighHorg sensitivity simulation.

The reduction in emission sources of NO*_x_* and SO_2_ impacted aerosol composition and thus the *T*_g,org_. The average *T*_g,org_ for the Emissions Reduction simulation predicted a small but statistically significant (*p* value = 2 × 10^−16^) increase of 1.5 K from the PhaseSep simulation, indicating the future emission reductions could result in minor increases in viscosity and frequency of phase separation. The overall phase-separation frequency for this sensitivity was 70.5% (57.0% SSPS, 13.5% LLPS), with predicted viscosities ranging from 6.13 × 10^−3^ to 1.73 × 10^11^ Pa s, which was slightly narrower compared to the *η*_org_ range from the PhaseSep simulation (refer to Sect. 3.1.1). With the implementation of the future reduced NO*_x_* and SO_2_ emissions, overall, there was a mean 7.85 % reduction in biogenic SOA at the surface level from the PhaseSep simulation for the continental United States. As shown in Fig. 10a, the areas with the largest reductions in SOA mass occurred in the American southeast, while a marginal increase in SOA mass that occurred over the Atlantic Ocean and in some sparse areas in northern Canada and the western United States. The American southeast was highly sensitive to the Emissions Reduction sensitivity due to the high concentrations of SO_2_ from coal-fired power plants and the high concentrations of IEPOX-derived SOA, whose chemistry is driven by particulate sulfate. Figure S5 shows that highest reductions in particulate sulfate occurs in the American southeast, accompanied by a reduction in aerosol liquid water. This drives the reductions in IEPOX-derived SOA as shown in recent literature (Pye et al., 2017) and hence the large reductions in biogenic SOA mass. Also, NO*_x_* reductions in the NO*_x_*-limited southeastern United States region essentially result in a larger decrease in biogenic SOA, as shown in Fig. 10a, which is consistent with findings from SENEX aircraft (Edwards et al., 2017) and SOAS ground measurements (Xu et al., 2015) in the southeastern United States.

When increases in *H*_org_ were simulated in the HighHorg scenario, it had impacts in opposite directions compared to changes in the Emissions Reduction scenario. This increase in biogenic SOA can simply be attributed to the increased dissolution of IEPOX into the particle phase through the organic coating with a *H*_org_ value 3 orders of magnitude higher than that in the PhaseSep simulation. The average *T*_g,org_ in the HighHorg simulation had a similarly small but also statistically significant increase of 1.4K, with particles being phase separated 68.3% of the time (55.8% SSPS, 12.5% LLPS). Predicted viscosities in this simulation were comparable to the PhaseSep simulation and ranged from 5.94 × 10^−3^ to 6.10^11^ Pa s. Overall, biogenic SOA mass increased by an average of 14.19% at the surface level for this simulation relative to PhaseSep for the continental United States. As shown in Fig. 10b, the regions with the largest increases in biogenic SOA mass were located over boreal forests in Ontario and Quebec, Canada, that correspond to the regions with highest reactive uptake (Fig. 7b and c) forming more homogeneous SOA with increased *H*_org_.

While modest improvement in model performance of PM_2.5_ OC by the aforementioned sensitivity simulations (HighHorg and PhaseSep2; see Sect. 3.2.2: “Comparison to observed data”) occurs, it does not addresses other major issues in the base CMAQ model performance. Firstly, these updates to phase state and phase-separation considerations only translate to IEPOX SOA, the only explicit parametrization of multiphase reactive uptake in CMAQ. IEPOX SOA alsojust makes up approximately 12% of the total PM_2.5_ OC mass simulated by CMAQ on an average for the 2013 SOAS period. There are other more important factors introducing major source of uncertainty in models across spatial scales including CMAQ, a very prominent uncertainty being missing representation of species like ONs reported as dominant in the 2013 SOAS by new instrumentation providing higher molecular detail (B. H. Lee et al., 2016; Massoli et al., 2018). Furthermore, field or laboratory studies on a wider suite of SOAs are needed to explicitly parametrize their multiphase chemistry and are still missing in CMAQ. It is a challenge to implement these mechanistic representations of different SOA holistically, without increased computational cost in CMAQ.

## 4 Discussion and atmospheric implications

Current chemical transport models have not accurately accounted for the effects of aerosol composition on phase separation or viscosity. This work has updated the CMAQ model to include parameters to calculate the *T*_g,org_ based on the Gordon–Taylor equation for SOA. This implementation used molar mass and O : C ratio of the species, but other parameters couldbe used. For example, DeRieux et al. (2018) developed a calculation for *T_g,i_* based on the number of carbon–hydrogen and carbon–oxygen bonds in a molecule. DeRieux et al. (2018) showed their implementation to be in good agreement with implementation provided in this work (Eq. 4) for species with molar masses in the range of those used by CMAQ v5.2.1. This implementation also included parameters to determine whether SOA was phase separated based on its viscosity, O : C ratio, sulfate concentrations and the ambient RH. Our updated PhaseSep2 model predicted up to 65.4% of the time particles would exhibit phase separation at the surface layer, which is in proximity with the ~ 70% predicted by Pye et al. (2017) for the isoprene-rich Centerville site in the southeastern United States. PhaseSep overestimated this phase-separation frequency at ~ 79.3%, indicating PhaseSep2 as a broader and accurate scenario for future implementations as well. This implementation predicts that most of the SOA in the middle and upper troposphere over the United States is phase separated with more organics in a semi-solid or even glassy state with increasing altitude. This is in agreement with previous fieldwork and modeling studies which have found that SOA in the upper troposphere tends to be in a glassy state (Lienhard et al., 2015; Shiraiwa et al., 2017). This work also shows LLPS to be more dominant in the eastern US, with the semi-solid phase state being more prevalent in the western US. This is in agreement with the predominant role of aerosol liquid water driving the liquid phase state and LLPS across the eastern United States, as observed in previous studies (Pye et al., 2017, 2018). The factors driving LLPS and SSPS are also an area that should be further studied due to the fact that the modifying the conditions for LLPS and SSPS led to large differences in IEPOXSOA.

The model predicted that SOA dominated by anthropogenic constituents typically featured thick semi-solid organic coatings surrounding aqueous cores, which caused the reactive uptake of IEPOX to become diffusion limited. Regions that were predicted to have larger fractions of biogenic SOA mass typically featured LLPS morphology that did not produce much of diffusion limitations. These aerosols also resulted in a smaller inorganic core volume increasing the concentrations of nucleophiles and acids, thus enhancing the rate of reaction in presence of abundant aerosol water over oceans but exhibited reduction in SOA over land, though not as much as solid-like particles exhibited. The phase-separation parameters had the largest impact over the Ohio River valley, southern Canada (more pronounced near Great Lakes) and the American southeast. These areas were also the most sensitive to future emission reductions of NO*_x_* and SO_2_.

Further experimental and modeling work is required to understand the effects of aerosol phase state on the viscosity of the inorganic core that cause variability in the value of *D*_a_ and can subsequently alter the reactive uptake of IEPOX. The combined effect of aerosol acidity and aforementioned higher core viscosity because of IEPOX SOA formation that has a self-limiting impact on IEPOX reactive uptake is also a caveat to be explored further (Zhang et al., 2019). The conditions under which highly viscous SOA will separate from inorganics in a particle or if the particle will remain homogeneously mixed should be further explored as well, given the differences in the frequency of predicted particle-phase separation between the PhaseSep and PhaseSep2 simulations and the implications that this has for the reactive uptake of IEPOX (Table S1). Constraining the viscosity of SOA in low RH (< 30 %) conditions is also an area that should be further explored to improve model performance. Furthermore, particle morphology in the event of phase-separated organic and inorganic species as “core–shell”, “partially engulfed” or “emulsified” (smaller islands of organics in the aqueous inorganic core) is driven by the differences in the interfacial surface tensions (Gorkowski et al., 2017). However, developing a computationally efficient method of modeling these alternative particle morphologies in CTMs is an area of ongoing research and needs further exploration. Recent studies have also shown that at very high RH ranges (95 %–100 %), some particles will return to a core–shell morphology (Ham et al., 2019; Renbaum-Wolff et al., 2016). There is also little information on the criteria that drive a particle to adopt a phase-separated morphology under these conditions. Such variability in particle morphologies may modify the value of *k*_particle_ by changing the core volume. It is imperative that these parameters be better constrained in models. Furthermore, there is much uncertainty in the organic shell Henry’s law coefficient (*H*_org_), where higher *H*_org_ increases the dissolution of IEPOX into the aerosol. Some of the newly proposed reaction mechanisms leading to the formation of extremely low-volatile organic compounds (ELVOCs) and organosulfates may also increase the viscosity of the particle but have not been incorporated into this study.

This work paves the way for implementing a more accurate representation of multiphase chemistry of different complex systems on the lines of explicit representation of IEPOX SOA. Multiphase chemistry of other dominant SOA apart from IEPOX SOA, such as monoterpene-derived SOA and ONs derived from both isoprene and monoterpenes, is not incorporated in CMAQ v5.2.1 (Pye et al., 2018; Slade et al., 2019) and should be a focus of future work. This work showed that organic water fraction is the biggest driver of viscosity, though the water abundance was set at a constant 10 % of the inorganic water content to better reflect observed concentrations of organic water during daylight hours relevant to IEPOX SOA chemistry. Organic water uptake, even if higher than the amount assumed here, will still follow the diurnal trend of RH since it is diverted from the aqueous core that is derived from ISORROPIA-based aerosol water in CMAQ v5.2.1. In this work, the O : C ratio of individual organic constituents as listed in Table 1 was used to calculate *T*_g,org_ based on Shiraiwa et al. (2017). The O : C provides an indication of hygroscopicity of different organic species (Pye et al., 2017), but it is only a surrogate. Lack of explicitly representing hydrophobicity or hygroscopic growth of various organic constituents is a limitation in the CMAQ modeling framework that was used. More recently, the degree of diffusivity or uptake of semi-volatile organic compounds (SVOCs) such as isoprene oxidation products and ONs into more viscous or semi-solid particle phase is found to differ. These changes in *η*_org_ profoundly impact both aerosol growth kinetics and their size distribution dynamics (Vander Wall et al., 2020; Zaveri et al., 2020). To assess the actual impacts of aging and hygroscopic growth under varying conditions, updates in the CMAQ model are required by adding explicit reactive uptake mechanisms for a wider range of non-IEPOX SOAs.

Performing sensitivity simulations in terms of different assumptions made on determining phase separation or morphology (PhaseSep2 and PhaseSep) is as important as constraining the *H*_org_ (HighHorg) factor in the regions with abundant IEPOX-SOA such as the American and Canadian southeast. Incorporating explicit kinetics coupled with thermodynamic calculation of energies governing the mixing state of organic–inorganic aerosol mixtures under different aerosol phase states, as observed from recent and ongoing experimental findings, into atmospheric models such as CMAQ, would lead to more scientifically sound representations of the impact particle-phase state and morphology have on SOA mass predictions.

## Supplementary Material

Supplement1

## Figures and Tables

**Table 1.** CMAQ-defined aerosol phase species (Pye et al., 2017; Murphy et al., 2017) used in the calculation of the predicted organic-phase parameter (overall SOA viscosity *η*_org_) and their respective organic mass to organic carbon ratio (OM : OC) (Pye et al., 2017), atomic oxygen to carbon ratio (O : C), molar weight (Pye et al., 2017) and predicted individual glass transition temperature (*T*_g_) and viscosity at standard temperature.

Species name	Description	Source	OM : OC ratio	O : C ratio	Molar weight (g mole^−1^)	Predicted *T*_g_(K)	Predicted *η*_org_ at *T* = 298 K and ALW = 0(Pa s)
AALK1	SV alkane VOC SOA	ANTH	1.56	0.315	225	256	7.54 × 10^9^
AALK2	SV alkane VOC SOA	ANTH	1.42	0.203	205.1	233	5.34 × 10^7^
ABNZ1	SV high-NO_*x*_ SOA product from benzene	ANTH	2.68	1.211	161	289	1.67 × 10^14^
ABNZ2	SV high-NO_*x*_ SOA product from benzene	ANTH	2.23	0.851	134	234	2.66 × 10^7^
ABNZ3	LV low-NO_*x*_ SOA product from benzene	ANTH	3.00	1.467	180	322	1.00 × 10^12^
AGLY	Glyoxal/methylglyoxal SOA	BIOG	2.13	0.771	66.4	160	1.71 × 10^3^
AISO1	SV SOA product from isoprene	BIOG	2.20	0.827	132.0	230	1.20 × 10^7^
AISO2	HV SOA product from isoprene	BIOG	2.23	0.851	133.0	233	2.26 × 10^7^
AISO3	Acid-catalyzed isoprene SOA compounds (2-methyltetrols plus IEPOX organosulfate)	BIOG	2.80	1.307	168.2	301	1.00 × 10^12^
ALVOO1	LV oxidized combustion organic compounds	ANTH	2.27	0.883	136	238	6.59 × 10^7^
ALVOO2	LV oxidized combustion organic compounds	ANTH	2.06	0.715	136	222	3.97 × 10^6^
AOLGA	Oligomer products of anthropogenic SOA compounds	ANTH	2.50	1.067	206	303	1.00 × 10^12^
AOLGB	Oligomer products of biogenic SOA compounds	BIOG	2.10	0.747	248	300	1.00 × 10^12^
AORGC	Glyoxal and methylglyoxal SOA	BIOG	2.00	0.667	177	251	1.33 × 10^9^
APAH1	SV high-NO_*x*_ SOA product from PAHs	ANTH	1.63	0.371	195.6	239	1.58 × 10^8^
APAH2	SV high-NO_*x*_ SOA product from PAHs	ANTH	1.49	0.259	178.7	216	2.80 × 10^6^
APAH3	LV low-NO_*x*_ SOA product from PAHs	ANTH	1.77	0.483	212.2	260	1.97 × 10^10^
APCSO	Potential combustion SOA	ANTH	2.00	0.667	170	245	3.91 × 10^8^
ASQT	SV SOA from sesquiterpenes	BIOG	1.52	0.283	135	179	1.87 × 10^4^
ASVOO1	SV oxidized combustion organic products	ANTH	1.88	0.571	135	207	4.69 × 10^5^
ASVOO2	SV oxidized combustion organic products	ANTH	1.73	0.451	135	195	1.10 × 10^5^
ASVOO3	SV oxidized combustion organic compounds	ANTH	1.60	0.347	134	184	3.19 × 10^4^
AIVPO1	Intermediate-volatility primary organic compounds	ANTH	1.17	0.003	266	260	3.22 × 10^10^
ALVPO1	LV primary organic compounds	ANTH	1.39	0.179	218	241	2.58 × 10^8^
ASVPO1	SV primary organic compounds	ANTH	1.32	0.123	230	245	7.00 × 10^8^
ASVPO2	SV primary organic compounds	ANTH	1.26	0.075	241	249	1.86 × 10^9^
ASVPO3	SV primary organic compounds	ANTH	1.21	0.035	253	254	6.63 × 10^9^
ATOL1	SV high-NO_*x*_ toluene SOA	ANTH	2.26	0.875	163	259	8.17 × 10^9^
ATOL2	SV high-NO_*x*_ toluene SOA	ANTH	1.82	0.523	175	23	7.25 × 10^7^
ATOL3	LV low-NO_*x*_ toluene SOA	ANTH	2.70	1.227	194	309	1.00 × 10^12^
ATRP1	SV SOA product from monoterpenes	BIOG	1.84	0.539	177	239	1.30 × 10^8^
ATRP2	HV SOA product from monoterpenes	BIOG	1.83	0.531	198	254	3.93 × 10^9^
AXYL1	SV high-NO_*x*_ SOA product from xylene	ANTH	2.42	1.003	174	278	3.16 × 10^12^
AXYL2	SV high-NO_*x*_ SOA product from xylene	ANTH	1.93	0.611	185	252	1.85 × 10^9^
AXYL3	LV low-NO_*x*_ SOA product from xylene	ANTH	2.30	0.907	218	297	1.43 × 10^16^

**Table 2.** Rate constants used to calculate the effective first-order rate constant for aqueous-phase IEPOX SOA formation catalyzed by H+, $\text{HS}{\text{O}}_{4}^{-}$ with water and $\text{S}{\text{O}}_{4}^{2-}$ as nucleophiles.

Rate constant	Value (M^−2^ s^−1^)	Reference
^*k*^H^+^, water	9.00 × 10^−4^	Eddingsaas et al. (2010), Pye et al. (2013)
$k_{\text{HS}{\text{O}}_{4}^{-}}$, water	1.31 × 10^−5^	Eddingsaas et al. (2010)
*k*_H^+^_,$\text{S}{\text{O}}_{4}^{2-}$	1.27×10^−3^	Riedel et al. (2016), Budisulistiorini et al. (2017)

**Table 3.** Brief summary of different simulations conducted in this work in CMAQ v5.2.1.

Simulations	Details
NonPhaseSep	Base CMAQ v5.2.1 parameterization assuming homogeneous, internally mixed organic-inorganic fine aerosol, no phase separation (Pye et al., 2017)

PhaseSep2	Additional term in reactive uptake calculation to capture the impact of phase separation and organic coating described in Sect. 2.1–2.3 and 2.5; Zuend and Seinfeld (2012) phase-separation criteria for both liquid and solid or semi-solid particles (see Sect. 2.3)

Emissions Reduction	EPA-recommended emission reductions for the year 2025 of 34% and 48 % for NO_x_ and SO_2_ from base 2013 scenario (see Sect. 2.6)

HighHorg	Higher organic-phase Henry’s law coefficient (3 orders of magnitude higher than in PhaseSep or PhaseSep2) (see Sect. 2.6)

PhaseSep	All parametrization is the same as in PhaseSep2 except for the sensitivity that Zuend and Seinfeld (2012) phase-separation criteria are not followed for solid or semi-solid particles (see Sect. 2.6)
