# Supplementary material for: Predicting secondary organic aerosol phase state and viscosity and its effect on multiphase chemistry in a regional-scale air quality model
Source: Atmos Chem Phys. Author manuscript; Available in PMC 2021 Jul 16. (PMC7510956; doi:10.5194/acp-20-8201-2020)
Supplement: Supplement1 [file NIHMS1623216-supplement-Supplement1.pdf]

Supplement of Atmos. Chem. Phys., 20, 8201–8225, 2020
https://doi.org/10.5194/acp-20-8201-2020-supplement
© Author(s) 2020. This work is distributed under
the Creative Commons Attribution 4.0 License.

Supplement of

Predicting secondary organic aerosol phase state and viscosity and its effect on multiphase chemistry in a regional-scale air quality model

Ryan Schmedding et al.

Correspondence to: William Vizuete (vizuete@unc.edu)

The copyright of individual parts of the supplement might differ from the CC BY 4.0 License.

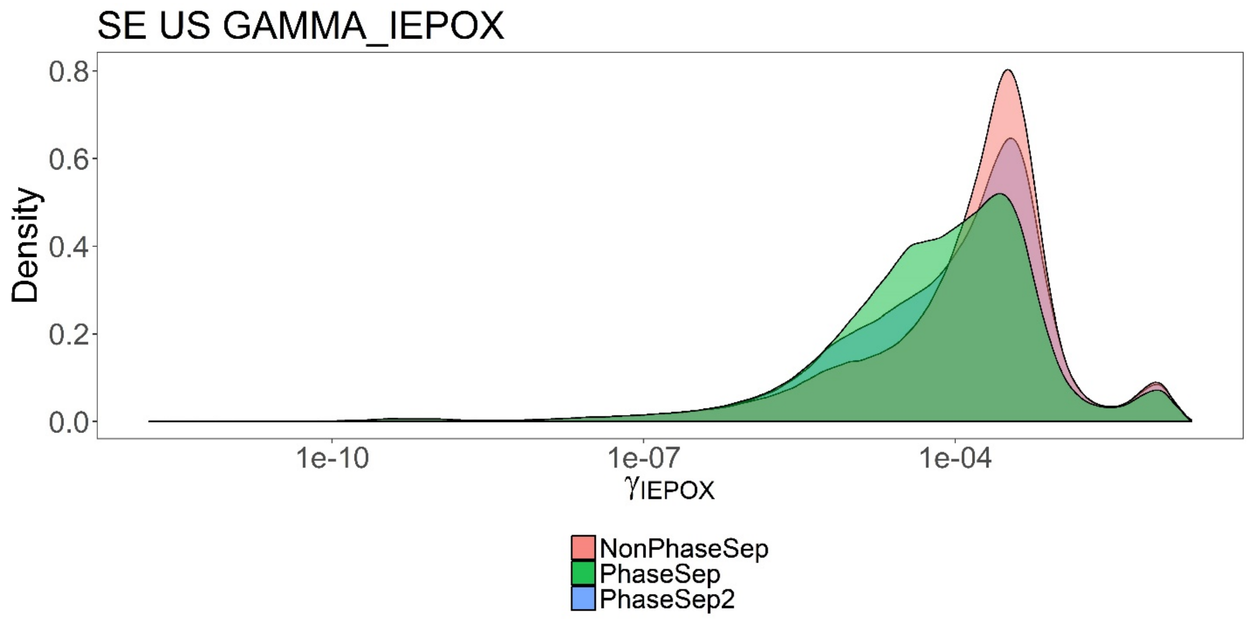

Figure S1 – For Southeastern United States, Probability distribution of γ_{IEPOX} at the surface level for the *NonPhaseSep* (red), *PhaseSep* (green) and *PhaseSep2* (blue) for SOAS 2013 simulation period.

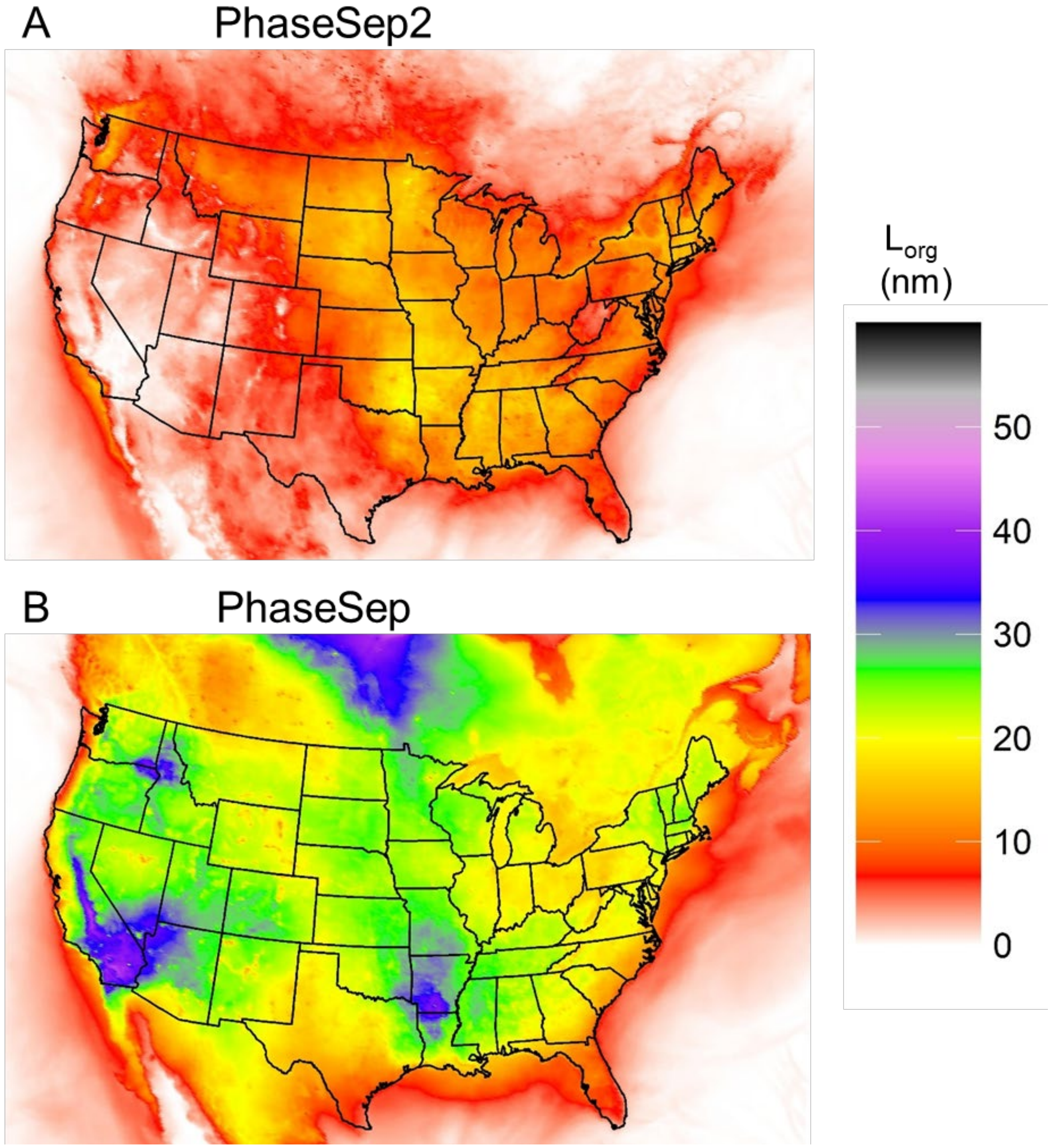

Figure S2 – Average organic coating thickness (l_{org} in nm) at the surface level for **(A)** *PhaseSep2* and **(B)** *PhaseSep* cases for SOAS 2013 simulation period.

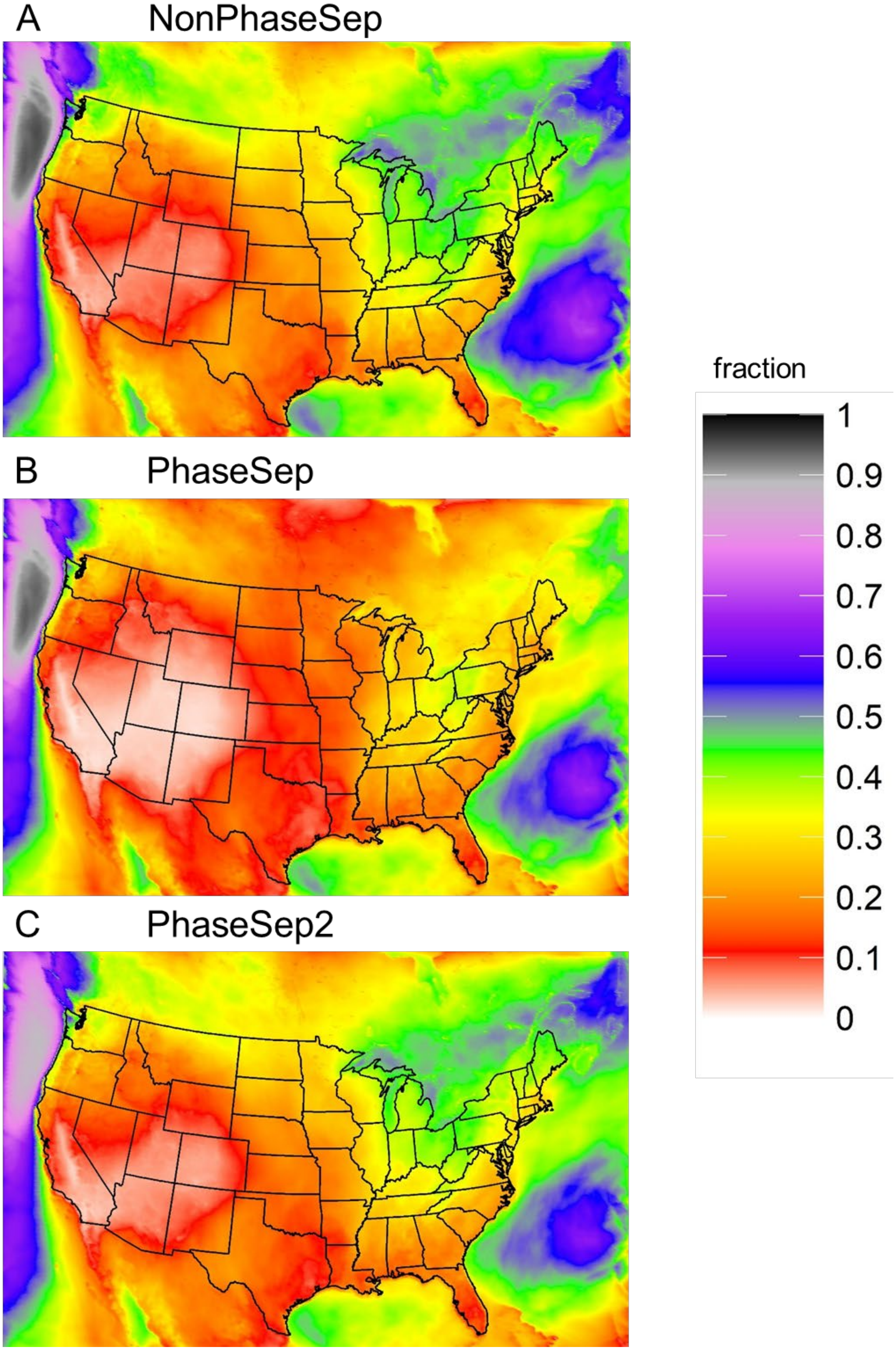
A
NonPhaseSep
B
PhaseSep
C
PhaseSep2
fraction
1
0.9
0.8
0.7
0.6
0.5
0.4
0.3
0.2
0.1
0

Figure S3 – Average fraction of IEPOX-derived SOA in biogenic SOA mass at the surface level for: **(A)** *NonPhaseSep*, **(B)** *PhaseSep* and **(C)** *PhaseSep2* case for SOAS 2013 simulation period.

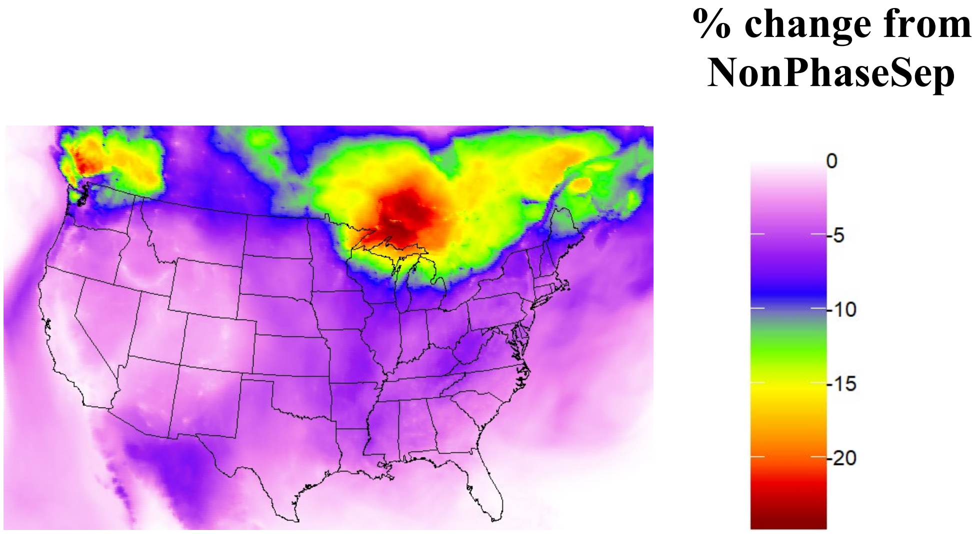

Figure S4 – Spatial map of the mean percent relative change of surface $PM_{2.5}$ organic carbon (OC) mass in *PhaseSep* case relative to the *NonPhaseSep* Simulation.

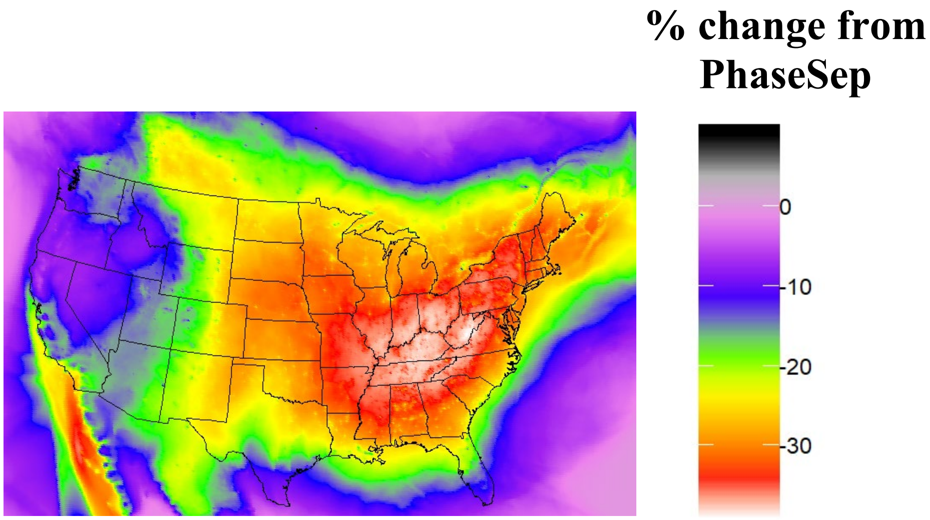

Figure S5 – Spatial map of the mean percent relative change of $PM_{2.5}$ sulfate mass at surface in *Emission Reduction* sensitivity case relative to the *PhaseSep* Simulation.

Table S1 – Comparison of different simulations on phase separation frequencies (for Continental US) and model performance in isoprene-abundant southeastern United States (rural Centreville, AL and urban Atlanta sites).

**Parameter** (units)		*PhaseSep*	*PhaseSep2*	*Emissions Reductions*	*HighHorg*
**For Continental US**	**LLPS Frequency** (%)	13.7	13.7	13.5	12.5
	**SSPS Frequency** (%)	54.8	29	57	55.8
**NMB** (%, compared to SEARCH $PM_{2.5}$ OC)	**Rural Centreville, AL forest site**	-36	-33	-44	-32
	**Urban Jefferson Street, Atlanta site**	-21	-18	-29	-18
